# Extending and improving metagenomic taxonomic profiling with uncharacterized species using MetaPhlAn 4

**DOI:** 10.1038/s41587-023-01688-w

**Published:** 2023-02-23

**Authors:** Aitor Blanco-Míguez, Francesco Beghini, Fabio Cumbo, Lauren J. McIver, Kelsey N. Thompson, Moreno Zolfo, Paolo Manghi, Leonard Dubois, Kun D. Huang, Andrew Maltez Thomas, William A. Nickols, Gianmarco Piccinno, Elisa Piperni, Michal Punčochář, Mireia Valles-Colomer, Adrian Tett, Francesca Giordano, Richard Davies, Jonathan Wolf, Sarah E. Berry, Tim D. Spector, Eric A. Franzosa, Edoardo Pasolli, Francesco Asnicar, Curtis Huttenhower, Nicola Segata

**Affiliations:** 1 Department CIBIO, University of Trento, Trento, Italy; 2 Harvard T.H. Chan School of Public Health, Boston, MA USA; 3 The Broad Institute of MIT and Harvard, Cambridge, MA USA; 4 IEO, European Institute of Oncology IRCCS, Milan, Italy; 5 Centre for Microbiology and Environmental Systems Science, University of Vienna, Vienna, Austria; 6 Zoe Global, London, UK; 7 Department of Nutritional Sciences, King’s College London, London, UK; 8 Department of Twin Research, King’s College London, London, UK; 9 Department of Agricultural Sciences, University of Naples, Naples, Italy

**Keywords:** Data processing, Metagenomics

## Abstract

Metagenomic assembly enables new organism discovery from microbial communities, but it can only capture few abundant organisms from most metagenomes. Here we present MetaPhlAn 4, which integrates information from metagenome assemblies and microbial isolate genomes for more comprehensive metagenomic taxonomic profiling. From a curated collection of 1.01 M prokaryotic reference and metagenome-assembled genomes, we define unique marker genes for 26,970 species-level genome bins, 4,992 of them taxonomically unidentified at the species level. MetaPhlAn 4 explains ~20% more reads in most international human gut microbiomes and >40% in less-characterized environments such as the rumen microbiome and proves more accurate than available alternatives on synthetic evaluations while also reliably quantifying organisms with no cultured isolates. Application of the method to >24,500 metagenomes highlights previously undetected species to be strong biomarkers for host conditions and lifestyles in human and mouse microbiomes and shows that even previously uncharacterized species can be genetically profiled at the resolution of single microbial strains.

## Abstract (web summary)

Integration of metagenomic assemblies and microbial isolate genomes improves profiling of uncharacterized species.

## Main

Over the last 25 years, shotgun metagenomic sequencing^1^ and associated computational methods have developed as robust, efficient ways to study the taxonomic composition^2–6^ and functional potential^4,7,8^ of complex microbial communities populating human, animal and natural environments. Genome assembly methods developed for microbial isolates have been expanded to apply to shotgun metagenomes, but while they excel in identifying new organisms from communities, their sensitivity is often limited by such environments’ complexity^9^. Reference-based computational approaches complement assembly by relying on annotated reference sequence information to accurately identify and quantify the known taxa and genes present in a microbiome by homology instead^4–7^. This set of methods enabled deep exploration of human microbiomes and the discovery of microbial associations with multiple health conditions^10–18^ and dietary patterns^19–23^, as well as the characterization of the evolution and transmission of microbial species and strains^24–29^. However, reference-based methods can only detect cataloged microbial species included in available reference databases, which typically only represent a fraction of the community members across environments, thus limiting the interpretation of shotgun metagenomes^30^.

Conversely, de novo metagenomic assembly to reconstruct draft genes and genomes—called metagenome-assembled genomes (MAGs)—has advanced to the point of very high specificity (albeit often low sensitivity) for recovery directly from metagenomes^31–35^. This allows recovery of microbial sequences that have not yet been isolated or characterized and are thus absent from reference databases^36^. As metagenomic assembly and binning have improved dramatically in the last few years^31–35^, large-scale MAG catalogs have been compiled and comprise a vast amount of unknown and uncultivated microbial species populating diverse environments^37–46^. However, such metagenomic assembly techniques are typically able to capture only a limited fraction of the organisms in complex communities due to insufficient coverage for many taxa, the presence of genetically related taxa impeding or creating spurious assemblies and difficulties in quality control of the resulting MAGs^9^.

To leverage the best aspects of both reference- and assembly-based metagenome profiling, we present MetaPhlAn 4, a method that exploits an integrated extended compendium of microbial genomes and MAGs to define an expanded set of species-level genome bins^42^ (SGBs) and accurately profile their presence and abundance in metagenomes. SGBs represent both existing species (known or kSGBs) or yet-to-be-characterized species (unknown, uSGBs) defined solely based on the MAGs^42^. From a collection of 1.01 M bacterial and archeal MAGs and isolate genomes integrating the most recent genome catalogs^37–45^ and additional newly assembled MAGs spanning multiple environments, we first expanded the definition of 54,596 SGBs and then defined SGB-specific unique marker genes (that is, genes uniquely characterizing each SGB) for 21,978 kSGBs and 4,992 uSGBs. The resulting dataset expands the existing MetaPhlAn algorithm^2–4^ to enable deeper and more accurate quantitative taxonomic analyses of human, host associated and environmental microbiomes and provides insights into a number of studies associating the microbiome with host conditions.

## Results

### MetaPhlAn 4 profiling of species-level genome bins

MetaPhlAn 4 expands and improves existing capabilities to perform taxonomic profiling of metagenomes by exploiting a framework in which extensive metagenomic assemblies are integrated with existing bacterial and archaeal reference genomes. These are then jointly preprocessed to allow efficient metagenome mapping against millions of unique marker genes, ultimately quantifying both isolated and metagenomically assembled organisms in new communities. The algorithm augments that used by previous versions in four main ways as follows: (1) the adoption of SGBs^42^ as primary taxonomic units, each of which groups microbial genomes and MAGs into consistent existing species and newly defined genome clusters of roughly species-level diversity; (2) the integration of over 1 M MAGs and genomes into this SGB structure to build one of the largest databases of confident microbial reference sequences currently available; (3) the curation of microbial taxonomic units based on the consistency of taxonomically labeled microbial genomes and the assignment of new taxonomic labels to SGBs solely defined on MAGs and (4) the improved procedure to extract unique marker genes out of each SGB for the MetaPhlAn reference-based mapping strategy^2–4^. MetaPhlAn 4 thus leverages aspects of both metagenomic assembly, with its potential to uncover previously unseen taxa^40–42,45^ and the sensitivity of reference-based profiling to provide accurate taxonomic identification and quantification.

The adoption of SGBs as the primary unit of taxonomic analysis is central to this approach^42^. Briefly, an SGB^42^ delineates a microbial species purely based on the clustering of whole-genome genetic distances at 5% genomic identity^47^ and a taxonomic label can then be assigned to the SGB based on the presence (or not) of characterized genomes from isolate sequencing. This definition permits arbitrary microbial genomes to be organized in a manner not unlike amplicons into operational taxonomic units (OTUs) and matches remarkably well the expected boundaries of the existing taxonomy^42,47,48^. Available microbial reference genomes and medium-to-high-quality MAGs are thus grouped into taxonomically well-defined species (‘known’ SGBs or kSGBs when an isolate genome with available taxonomy is present in the SGB) or unknown equivalent clades (uSGBs).

Following the SGB clustering approach, the database employed by MetaPhlAn 4 contains SGBs that result from the merging of species that were originally incorrectly taxonomically labeled as separate species. For example, genomes assigned in NCBI^49^ to *Lawsonibacter asaccharolyticus* and *Clostridium phoceensis* are 98.7% identical, likely due to independent naming of members of a new species and were merged into the SGB15154 (Supplementary Table 1). This merging also applies to taxonomic species that are genetically difficult or impossible to distinguish (for example, species of the *Bacillus cereus* group, genetically differentiated only by their plasmidic sequences^50^) and are thus clustered in the same SGB. Conversely, species with subclades diverging for more than 5% genetic identity were split into multiple SGBs (for example, *Prevotella copri* is represented by four different SGBs^51^, or *Faecalibacterium prausnitzii* with SGBs representing its distinct (sub)species^52^; Supplementary Table 1). Finally, incorrectly or partially taxonomically classified reference genomes were detected and amended based on the detection of outlier labels resulting from misspellings or incorrect assignments by NCBI genome submitters (for example, the *Staphylococcus epidermidis* SGB7865 is composed of 700 reference genomes, 32 of which have different or unspecified species labels in the NCBI database^49^, Supplementary Table 1).

**Fig. 1:**
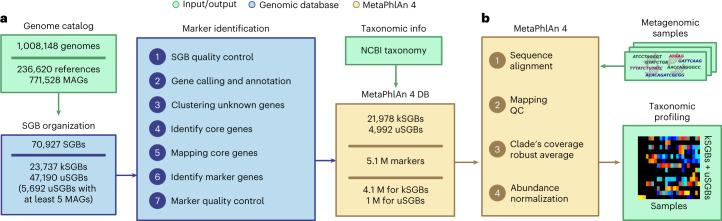
MetaPhlAn 4 integrates reference sequences from isolate and metagenome-assembled genomes for metagenome taxonomic profiling. **a**, From a collection of 1.01 M bacterial and archeal reference genomes and metagenomic-assembled genomes (MAGs) spanning 70,927 species-level genome bins (SGBs), our pipeline defined 5.1 M unique SGB-specific marker genes that are used by MetaPhlAn 4 (avg., 189 ± 34 per SGB). **b**, The expanded marker database allows MetaPhlAn 4 to detect the presence and estimate the relative abundance of 26,970 SGBs, 4,992 of which are candidate species without reference sequences (uSGBs) defined by at least five MAGs. The profiling is performed firstly by (1) aligning the reads of input metagenomes against the markers database, then (2) discarding low-quality alignments and (3) calculating the robust average coverage of the markers in each SGB that (4) are normalized across SGBs to report the SGB relative abundances (see Methods). All data are presented as mean ± s.d.

To derive the database of SGBs to be profiled in MetaPhlAn 4, the isolate genome component included 236,620 bacterial and archeal genomes available in NCBI^53^ and labeled as ‘reconstructed from isolate sequencing or single cells’. These were integrated with 771,528 MAGs assembled from samples collected from humans (five distinct main human body sites, 164 distinct human cohorts), animal hosts (including 22 nonhuman primate species) and nonhost-associated environments (including soil, fresh water and oceans; Supplementary Tables 2 and 3). After removing reference genomes and MAGs that did not meet quality control criteria (that is, genome completeness above 50% and contamination below 5%; see Methods), the catalog comprised 729,195 genomes (560,084 MAGs and 169,111 reference genomes) and was Mash^54^ clustered into SGBs at 5% sequence similarity^42^ for the final database of 70.9 k SGBs, 47.6 k of which are taxonomically unknown at the species level (uSGBs; Fig. 1a). This catalog spans 95 different phyla that are quite consistently enriched by uSGBs (Supplementary Table 4). In comparison with the original SGB catalog^42^, the current collection integrates 3.6 times more MAGs from highly diverse environments (Supplementary Table 3) and resulted in the definition of 4.3 times more SGBs. While the repository can be used for genome-based studies at a larger scale than what has been described so far^40–42,45,51,55–57^, we focused here on the task of identification and quantification of taxa from metagenomes. To this end, and to decrease the potential rate of false-positive detection of SGBs without strong support or that are extremely rare, we retained only the uSGBs containing at least five MAGs from distinct samples for subsequent metagenome profiling, resulting in a final catalog of 29.4 k quality-controlled SGBs (see Methods).

From this SGB genome catalog, we built the pangenome of each SGB (collection of all gene families found in at least one genome in the SGB) and used them to identify species-specific marker genes for MetaPhlAn profiling. The pangenomes were built by categorizing the coding sequences of all the 729 k genomes into UniRef90 clusters^58^ when a 90% amino acid identity match was found within the UniRef database, or by de novo clustering all remaining sequences at 90% amino acid identity following the Uniclust90 criteria^59^ (see Methods). From the resulting 50.6 M UniRef90 identities and 77.7 M new Uniclust90 gene families, we subsequently identified core gene families (that is those present in almost all genomes and MAGs of an SGB; see Methods) and then screened them for their species-specificity by mapping against all sequences of all SGBs (see Methods). This procedure resulted in 5.1 M total unique marker genes spanning 26,970 high-quality SGBs, with an average of 189 ± 34 unique marker genes per SGB. MetaPhlAn 4 taxonomic profiling uses these markers to detect the presence of an SGB (known or unknown) in new metagenomes based on the detection via read mapping of a sufficient fraction of SGB-specific marker genes (default 20%) and quantifies their relative abundance based on the within-sample-normalized average coverage estimations (see Methods; Fig. 1b).

### MetaPhlAn 4 improves the performance of taxonomic profiling

To evaluate the taxonomic profiling performance of MetaPhlAn 4, we first assessed its ability to profile well-characterized species (that is, those belonging to kSGBs) in comparison with available methods by using 133 synthetic metagenomes (~4B total reads). Most of these synthetic samples (128) are from the CAMI 2 taxonomic profiling challenge^60^ representing host-associated and marine communities, whereas the other five are additional nonhuman synthetic metagenomes (derived from SynPhlAn; see Methods) representing more diverse environments than in previous evaluations^4^.

**Fig. 2:**
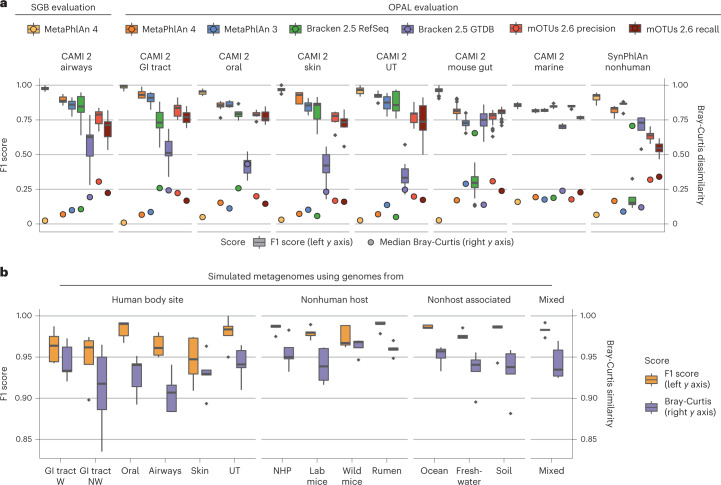
MetaPhlAn 4 improves sensitivity and specificity of metagenome taxonomic profiling. **a**, To evaluate its performance in taxonomic profiling, MetaPhlAn 4 was applied to synthetic metagenomes representing host-associated communities from the CAMI 2 taxonomic profiling challenge^60^ (*n* = 128 samples) and the SynPhlAn-nonhuman dataset (*n* = 5 samples), representing more diverse environments from previous evaluations^4^. Species-level evaluation using the OPAL framework^61^ shows that MetaPhlAn 4 is more accurate than the available alternatives in both the detection of which taxa are present (the F1 score is the harmonic mean of the precision and recall of detection) and their quantitative estimation (the BC beta-diversity is computed between the estimated profiles and the abundances in the gold standard). Additional evaluations performed using genomes within the SGB organization (labeled ‘SGB evaluation’; see Methods) show that MetaPhlAn 4 further improves accuracy at this more refined taxonomic level. See Supplementary Tables 5 and 7 for more details (GI, gastrointestinal; UT, urogenital tract). **b**, MetaPhlAn 4 was applied to synthetic metagenomes (*n* = 70 samples) modeling different host and nonhost-associated environments and containing, on average, 47 genomes from both kSGBs and uSGBs (see Methods). This evaluation directly on SGBs shows the reliability of MetaPhlAn 4 to quantify both known and unknown microbial species. Additional evaluation based on a mixture of new MAGs from samples not considered in the building of the genomic database (mixed evaluation, *n* = 5 samples) stresses its accuracy independently from the inclusion of the profiled data in the database. See Supplementary Tables 9 and 10 for more details (NHP = nonhuman primates, W = westernized, NW = nonwesternized). Box plots in **a** and **b** show the median (center), 25th/75th percentile (lower/upper hinges), 1.5× interquartile range (whiskers) and outliers (points).

Through the OPAL benchmarking framework^61^, we evaluated MetaPhlAn 4 in comparison with MetaPhlAn 3 (ref. ^4^), mOTUs 2.6 (ref. ^6^) (latest database available as for March 2021) and Bracken 2.5 (ref. ^5^) (with two databases, one built using the April 2019 RefSeq release^62^ and another one built using the GTDB release 207 (ref. ^63^)). Due to the high false-positive rates reported by Bracken 2.5, we decided to evaluate its performance by filtering out low-abundant hits (minimum relative abundance 0.01%; Supplementary Fig. 1). MetaPhlAn 4 outperformed the other tools when assessing the F1 score (Fig. 2a) computed based on the common reference NCBI taxonomy. This was true despite the fact that OPAL does not consider SGB-defined species groups (that is, single species incorrectly taxonomically labeled as separated species and included in the same SGB), thus penalizing MetaPhlAn 4 profiling that cannot match the corresponding labels; the new version still achieved a higher number of species correctly detected compared with MetaPhlAn 3 across all simulations (avg., 96.65 ± 66.08 and 85.32 ± 61.95 true positives, respectively) while maintaining a low number of false positives (avg., 16.09 ± 17.65 and 13.63 ± 16.56, respectively; Supplementary Fig. 2a,b and Supplementary Table 5). Most of the false positives (84.6%) were due to the new labels of SGB-defined species groups (for example, the *Marinilactibacillus* sp. 15R, present in almost all the CAMI 2 oral metagenomes, belongs to the *Marinilactibacillus piezotolerans* SGB7875 species group) and are thus also not strictly false positives. In fact, further evaluation using single isolate sequences (see Methods) showed no false-positive hits when running MetaPhlAn 4 with default parameters, and no false negatives in all cases with a coverage ≥ 0.5×. This coverage threshold means that MetaPhlAn 4 is guaranteed to detect all SGBs that are at a relative abundance of at least 0.01% for a metagenomic sample at a standard depth of 10Gbases with detection at lower abundances frequently possible (Supplementary Table 6). The improvement in recall is substantially explained by the expanded catalog of reference genomes included in MetaPhlAn 4 (169.1 k genomes spanning 31.9 k species in comparison with 99.2 k genomes from 13.5 k species in MetaPhlAn 3).

We then evaluated the relative abundance quantification performance of MetaPhlAn 4 using Bray-Curtis (BC) dissimilarity and root-mean-square error (RMSE) with respect to synthetic reference community compositions. MetaPhlAn 4 outperformed the alternative methods (avg. BC, 0.13 ± 0.07; avg. RMSE, 0.016 ± 0.019), including the previous MetaPhlAn version 3 (avg. BC, 0.19 ± 0.12; avg. RMSE, 0.019 ± 0.018; Supplementary Table 7 and Fig. 2a). The quality of the marker set is likely the driving factor of this improvement, a consequence of the phylogenetic consistency of the SGBs that ensures that identically-labeled taxa are genomically consistent. This avoids hard-to-detect taxonomic mislabeling in the original, manually assigned taxonomic labels and allowed us to obtain a set of marker genes that (1) is larger (avg., 189 ± 34 per SGB as compared to 84 ± 47 per species in MetaPhlAn 3), (2) more reliable (Supplementary Table 6 and Supplementary Fig. 3) and (3) more unique (99.3% of the markers in comparison with 72.7% in MetaPhlAn 3, and from 3.8× to 15.55× less randomly assigned reads depending on the environments; Supplementary Table 8).

Apropos, because these evaluations were not able to account for modifications of species taxonomy that avoid these issues, we then evaluated MetaPhlAn 4 on the same synthetic metagenomes, but using SGB-based taxonomy (see Methods). By considering as the gold standard label for each genome in the synthetic community the SGBs it belongs to, MetaPhlAn 4 achieved high accuracies when assessing both the F1 score (avg., 0.95 ± 0.06) and the BC dissimilarity (avg., 0.031 ± 0.023; Fig. 2a).

Finally, we assessed the performance of MetaPhlAn 4 to specifically detect uSGBs representing clades without taxonomically characterized isolates. We constructed 65 synthetic metagenomes simulating microbiomes from 12 different human body sites, animal hosts and nonhost-associated environments, using both kSGBs and uSGBs that were found and reconstructed in real metagenomes in each of the environments via metagenomic assembly (see Methods). We also built five additional synthetic metagenomes using a mixture of MAGs and reference genomes from samples not included in our original genomic database (see Methods). MetaPhlAn 4 showed accuracies in the detection and quantification of uSGBs (avg. F1 score, 0.97 ± 0.02; Fig. 2b and Supplementary Fig. 2c,d) that were on par with those of known species (kSGBs; avg. F1 score, 0.96 ± 0.024; Fig. 2a). Both the F1 score and the BC similarity to the gold standard were consistent across all the different environments assessed. Synthetic samples based on the MAGs not available at the time when the MetaPhlAn 4 database was built yielded similar results (avg. F1 score, 0.98 ± 0.006; Fig. 2b and Supplementary Tables 9 and 10). Altogether, MetaPhlAn 4 outperformed the other available tools on synthetic data and further provided quantification of yet-to-be-characterized species, while maintaining high accuracy for taxonomically well-defined species.

### MetaPhlAn 4 expands the profiled fraction of metagenomes

**Fig. 3:**
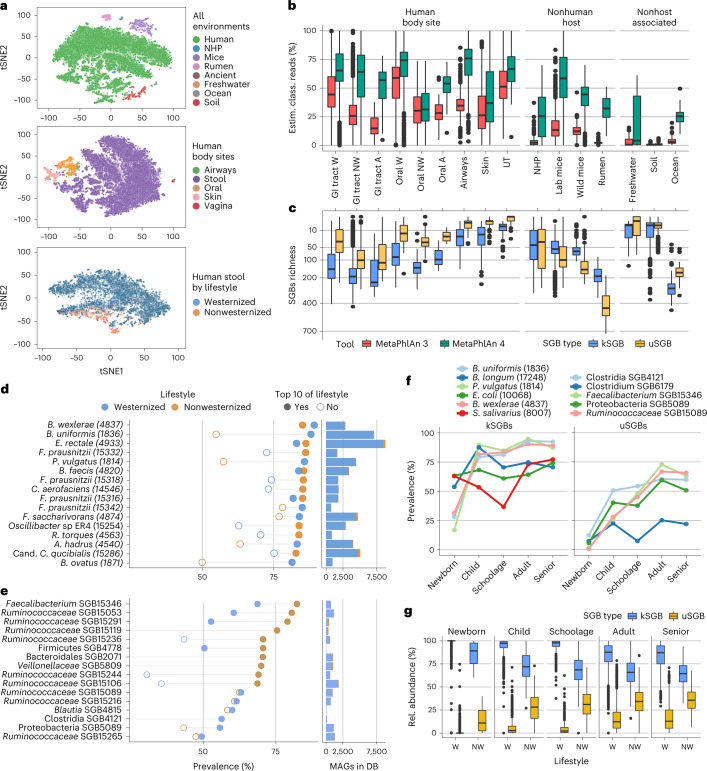
MetaPhlAn 4 expands observable microbial diversity, primarily by quantifying yet-to-be-characterized species (uSGBs). **a**, We applied MetaPhlAn 4 profiling to a total of 24.5 k metagenomic samples from diverse environments, highlighting its ability to detect microbiome compositions and clear differences between them, even when considering distinct human body sites and variable host lifestyles (Supplementary Fig. 5b and Supplementary Table 11). **b**, The expanded genomic database of MetaPhlAn 4 substantially increases the estimated fraction of classified reads in comparison with the previous MetaPhlAn version across habitat types (*n* = 24,515 samples). **c**, MetaPhlAn 4 detects on average 48 unknown bacterial species (uSGBs) per human gut microbiome, and reaches up to more than 700 in other nonhuman environments (*n* = 24,515 samples). **d**, The most prevalent microbial species in the gastrointestinal tract of westernized populations are known species (kSGBs). The ten most prevalent kSGBs in westernized and nonwesternized lifestyles are shown ordered by their highest prevalence and reported together with the number of MAGs assembled from human gut metagenomes in the MetaPhlAn genome catalog. Species names are shown together with their SGB ID between brackets. **e**, The most prevalent SGBs in nonwesternized populations belong to yet-to-be-cultivated and named species. The ten most prevalent uSGBs of each lifestyle are shown ordered by their highest prevalence. **f**, In westernized populations, the most prevalent kSGBs and uSGBs vary across age categories. The two most prevalent SGBs for each age category are shown. **g**, The fraction of uSGBs relative to kSGB increases after infancy (*n* = 19,468). Box plots in **b, c** and **g** show the median (center), 25th/75th percentile (lower/upper hinges), 1.5× interquartile range (whiskers) and outliers (points). NHP, nonhuman primates; W, westernized; NW, nonwesternized; A, ancient.

The MetaPhlAn 4 database expands the number of quantifiable known microbial species (18.4 k more species than in MetaPhlAn 3) and refines the resolution of many species described by kSGBs (21,978 kSGBs, with avg. 1.15 kSGBs per species), and includes 4,992 yet-to-be-characterized microbial species (uSGBs). We assessed its resulting increased ability to explain a larger fraction of the reads in a metagenome by profiling a total of 24.5 k metagenomic samples (145 distinct studies, Supplementary Table 11) from different human, animal and nonhost-associated environments (Fig. 3a and Supplementary Fig. 4). We further divided the 19.5 k human metagenomes based on the body site of origin and the lifestyle (that is, westernized or nonwesternized) of the donor (for a full description of westernization, see Methods).

In the resulting taxonomic profiles, MetaPhlAn 4 detected 11,132 SGBs present in at least 1% of the samples of one of the environments, 3,527 of which (31.68%) were taxonomically unknown at the species level (uSGBs). The new profiles explained a much larger fraction of the reads in the metagenomic samples compared to the previous version across all environments (Fig. 3b). Within the human body sites, the improvement was high for the airways (avg. 1.95-fold increase of explainable reads), and substantially higher improvements were reached for samples from, for example, the gut microbiomes of nonhuman mammals ranging from the average 3.26-fold increase of the wild mice to 14.15-fold increase in the rumen. For these animals, the average number of uSGBs detected surpassed that of the kSGBs (with the exception of the nonhuman primates; Fig. 3c and Supplementary Fig. 5a). These increases were consistent with the number of newly considered MAGs that defined new uSGBs from nonhuman microbiomes (90,606 MAGs defining 1,287 uSGBs).

Environmental ecosystems had metagenomes that were generally less explained by the taxa considered in MetaPhlAn 4, with soil, in particular, remaining poorly characterized due to its remarkable microbial variability and the lack of systematic large metagenomic efforts targeting it (only 2,495 MAGs defining 26 uSGBs in our database), while the ocean microbiome had a 6.65-fold increase, largely due to the inclusion of the Tara ocean MAGs^64^ in the SGB database (Fig. 3c). Overall, uSGBs were instrumental to increase the fraction of metagenomes profileable by MetaPhlAn 4 (Fig. 3b), as they accounted for an average of 23.13% (s.d.: 17.89%) of the richness of the resulting profiles across all environments (Fig. 3c).

### SGB profiling reveals species overlaps across environments

A key advantage of reference-based metagenomic profiling as compared to assembly is its ability to detect low-abundant and hard-to-assemble genomes^37,39,40,42,43,45^. This allows the generation of confident ecological statistics regarding prevalent and rare taxa, which are difficult to quantify accurately in the presence of many technical nondetections in data solely from metagenome assemblies. On this dataset, MetaPhlAn 4 identified 1,657 SGBs found in at least 1% of the samples from the gut of nonwesternized human populations (550 of these being uSGBs), 331 SGBs at the same prevalence threshold in the typically low-diverse human vaginal microbiome (61 of which are uSGBs) and intermediate numbers in other environments (Supplementary Fig. 5b).

This confirmed that gut metagenomes retrieved from ancient samples (ranging from 5,300 to 150 years ago in the available datasets) possessed more SGBs in common with those at >1% prevalence in the gut microbiome of modern nonwesternized populations (1,039 SGBs) than of westernized ones (748 SGBs), despite the dominance in datasets and databases of data derived from westernized populations (~ten times more samples; Supplementary Fig. 5b and Supplementary Table 3). Similarly, and adopting the same prevalence threshold at 1%, the SGBs found in the gut of nonhuman primates (including those in captivity) overlapped more with gut samples from ancient microbiomes (879 SGBs) than with modern ones (668 SGBs), further highlighting the effect of lifestyle in shaping the human microbiome (Supplementary Fig. 5b). A similar environmental adaptation can be observed in the gut microbiome of laboratory mice, in which many more modern human gut SGBs were found (481 SGBs) compared to those from wild mice (53 SGBs). Twenty-eight SGBs were present at >1% prevalence in all human body sites (Supplementary Table 12), comprising typically oral microbes that can reach the lower gastrointestinal tract, can contaminate the skin and can colonize other mucosal sites such as the vagina, that is, the *Haemophilus parainfluenzae* group (SGB9712), the *Streptococcus salivarius* group (SGB8007), *Veillonella parvula* (SGB6939), *Rothia mucilaginosa* (SGB16971) and *Streptococcus oralis* (SGB8130).

Species that overlap across environments at the same 1% prevalence threshold can also spot potential contamination as it is the case of the only nine SGBs shared between the modern human gut and ocean water samples (Supplementary Table 13). These were predominantly skin and oral microbes likely to contaminate low-biomass water samples during laboratory procedures as follows: *Cutibacterium acnes* (SGB16955), *Staphylococcus aureus* (SGB7852), *Streptococcus thermophilus* (SGB8002), *Escherichia coli* (SGB10068), *V. parvula* (SGB6939), *Staphylococcus epidermidis* (SGB7865), *Staphylococcus hominis* (SGB7858), *Streptococcus mitis* (SGB8163) and *R. mucilaginosa* (SGB16971). Overall, the new MetaPhlAn 4 profiling highlights that microbiomes from most nonhost-associated environments have little overlap between themselves and the human microbiome (Supplementary Fig. 5c), and that, as expected, human microbiomes from different body sites have limited but relevant overlaps (Supplementary Table 12).

### MetaPhlAn 4 expands the panel of prevalent human gut species

We assessed the prevalence of SGBs in the gut microbiome of human individuals (Supplementary Table 14) using 19.5 k human gut metagenomes from 86 datasets, spanning different age categories, geographic locations and lifestyles (Supplementary Table 15). The most prevalent SGBs in westernized populations were from known species (Fig. 3d), specifically *Blautia wexlerae* (SGB4837, 89.2%), the *Bacteroides uniformis* group (SGB1836, 88.1%) and *Phocaeicola vulgatus* (previously *Bacteroides vulgatus*, SGB1814, 85.8%). Four distinct *F. prausnitzii* SGBs appeared within the top ten most prevalent species, and three of them had quite distinct prevalence in both lifestyles (Fig. 3d), highlighting the ability of SGB profiling to increase the resolution of species that are particularly genetically divergent^52^. *Cibionibacter quicibialis*^42^, as well as several other species of interest considered kSGBs because they have a sequenced representative even though they remain largely uncharacterized (for example, *Oscillibacter sp*. ER4) were also found at high prevalence (Fig. 3d).

While most uSGBs had lower prevalence in this population, 4 uSGBs from the *Ruminococcaceae* family exceeded 75% prevalence, and many of them were substantially more prevalent in nonwesternized compared to westernized populations (Fig. 3e). The species with the highest prevalence in each specific age category displayed variable prevalence in the other age groups (Fig. 3f, Supplementary Fig. 6 and Supplementary Table 14), and uSGBs tended to be particularly common in childhood, which may be under-studied relative to infancy and adulthood (Fig. 3g and Supplementary Table 16). Overall, the newly established SGBs prevalence across population and lifestyles (Supplementary Table 14) expands both the size and detail of that established by prior metagenomic studies.

### Biomarkers of diet in mice are dominated by uSGBs

**Fig. 4:**
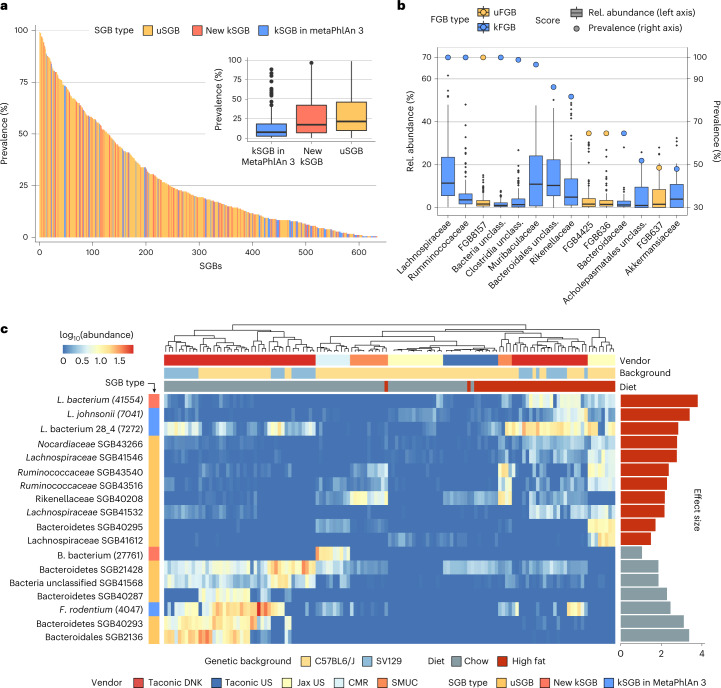
MetaPhlAn 4 enables accurate metagenomic profiling of mouse microbiomes containing few cultured isolate taxa. **a**, MetaPhlAn 4 taxonomic profiling of a cohort of mouse gut microbiome samples (*n* = 181 samples), spanning eight genetic backgrounds and six different vendors^65^ revealed that the majority of detected microbial taxa are uncharacterized SGBs (uSGBs) that do not contain a sequenced isolate representative. **b**, Some of the most prevalent families in the mouse gut microbiome (*n* = 181 samples) are still unclassified at the family level (uFGBs). FGBs detected in at least 20% of the samples (circles and right-side *y* axis) and with a median relative abundance above 1% (box plots and left-side *y* axis) are shown. **c**, Random effects models applied to the MetaPhlAn 4 profiles revealed that most of the high- and low-fat diet microbial biomarkers are uncharacterized species (FDR < 0.2). log_10_-transformed relative abundances of the microbial biomarkers are represented in the heatmap and their effect size (linear model beta coefficient) in the bar plots. For kSGBs, species names are shown together with their SGB ID between brackets. SGB41568 is reported in NCBI as assigned to an unclassified phylum, and we thus report only the kingdom label. SMUC = Southern Medical University in China, CMR = Craniofacial Mutant Resource at the Jackson Laboratory (Jax). Box plots in **a** and **b** show the median (center), 25th/75th percentile (lower/upper hinges), 1.5× interquartile range (whiskers) and outliers (points).

MetaPhlAn 4 integrates 22,718 MAGs assembled from 1,906 mouse gut metagenomes (both research laboratory mice and wild mice) and defines 540 uSGBs, allowing greater resolution in profiling the mouse gut. When applied to a heterogeneous public dataset of 184 mouse gut microbiomes spanning eight genetic backgrounds and six different vendors (Supplementary Table 17)^65^, MetaPhlAn 4 detected 632 different SGBs, 45.57% of them that would not be detected using only MAGs reconstructed from the same samples’ set (Supplementary Table 18). As already noted in recent studies^66^ employing a metagenomic-assembly-based workflow^67^, most of the detected SGBs in the mouse gut (60.8%) were uSGBs (Fig. 4a and Supplementary Fig. 7a). In contrast, only 108 total species were detected by MetaPhlAn 3 from the same samples. Interestingly, of the 43 SGBs present in more than 75% of the samples, most are uSGBs; the 12 kSGBs themselves represent poorly characterized species such as *Lachnospiraceae* bacterium 28_4 (SGB7272), *Dorea* sp. 5_2 (SGB7275) and *Oscillibacter* sp. 1_3 (SGB7266), which were also the only ones detectable by MetaPhlAn 3. The poor mappability of many mouse microbiomes against isolate genomes is also reflected at taxonomic levels higher than species, as more than half of the families (that is, family-level genome bins (FGBs) defined similarly to SGBs but spanning up to 30% nucleotide divergence; see Methods) present in more than 20% of the samples are still uncharacterized (uFGBs; Fig. 4b and Supplementary Fig. 7b).

To test the relevance of uSGBs in the context of typical mouse microbiome studies, we recapitulated prior statistical tests to identify taxonomic biomarkers of high-fat (HF) versus normal chow diets across host genetic backgrounds and vendors^65^. Applying linear mixed models on the MetaPhlAn 4 taxonomic profiles and controlling for sex, age, genetic background and vendor (see Methods; Supplementary Table 19), we identified 18 SGB biomarkers at FDR < 0.2 with an average relative abundance in the associated diet >1% (Fig. 4c). Most of the over-abundant biomarkers of a hyper-caloric diet were uSGBs (13 uSGBs, 72% of the 18 biomarkers), in addition to three taxa that could be detected using MetaPhlAn 3 (*Lachnospiraceae* bacterium 28_4 SGB7272, *Lactobacillus johnsonii* SGB7041 and *Faecalibaculum rodentium* SGB4047) and 2 kSGBs representing poorly characterized species (*Lachnospiraceae* bacterium SGB41544 and Bacteroidales bacterium SGB27761). While other approaches are already available to exploit environment-specific MAG catalogs for metagenomic profiling^67–69^, MetaPhlAn 4’s ability to rapidly and accurately profile species defined solely by MAGs (that is, uSGBs) appears particularly relevant for under-characterized microbial environments in which cultivated and sequenced taxa still represent a small fraction of overall microbial diversity.

### Stronger links between gut microbiome, diet and metabolism

**Fig. 5:**
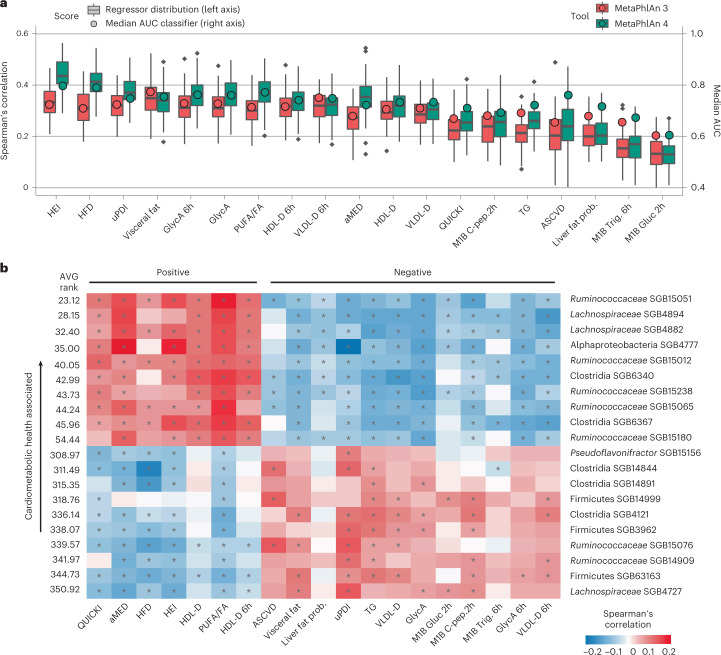
MetaPhlAn 4 reveals strong links between the unknown fraction of the human gut microbiome and host diet and cardiometabolic markers. **a**, Compared to the original results from the ZOE PREDICT 1 study based on the MetaPhlAn 3 taxonomic profiles^22^, random forest (RF) models trained on the MetaPhlAn 4 microbiome profiles (*n* = 1,001 samples) substantially improve classification (circles and right-side *y* axis) and regression (box plots and left-side *y* axis) result for a panel of 19 markers representative of nutritional and cardiometabolic health (see Methods). Box plots show the median (center), 25th/75th percentile (lower/upper hinges), 1.5× interquartile range (whiskers) and outliers (points.) **b**, Panel of the 20 unknown microbial species (uSGBs) showing the strongest overall correlations with the positive (top-half list) and negative (bottom-half list) dietary and cardiometabolic health markers, respectively (^∗^FDR < 0.2).

We used MetaPhlAn 4 to extend links between the gut microbiome, diet and host metabolism^19–23,70^ by re-analyzing metagenomes from 1,001 deeply phenotyped individuals in the ZOE PREDICT 1 study^22^. As in the original study, strengths of association between the microbiome and both dietary and cardiometabolic host variables were evaluated by testing the predictive power of random forest (RF) classifiers and regressors trained on the taxonomic profiles (see Methods). Among the 19 health and diet markers most strongly linked with the microbiome according to MetaPhlAn 3 in the original work, all but two were better predicted when incorporating MetaPhlAn 4 taxa (new median AUC = 0.74, 4.84% improvement; Fig. 5a). The highest improvement was found for the 10-year atherosclerotic cardiovascular disease (ASCVD) risk (0.106 higher AUC, 16.24% improvement), and the Healthy Eating Index (HEI) score^71^ achieved the strongest association (0.072 higher AUC, 10.05% improvement and 31% regression improvement).

Microbiome links with dietary indices were particularly improved by considering uSGBs (Fig. 5a); previously, visceral fat and blood lipid levels were generally more strongly microbiome-associated than dietary indices using MetaPhlAn 3 profiles. This was substantiated by the analysis of correlation between the abundance of each uSGB with all 19 host diet, anthropometric and physiology indices. Indeed, the strongest correlations (after accounting for age, sex and BMI; Fig. 5b) mostly involved uSGBs (6 of the 10 SGBs most associated with healthy conditions were uSGBs), and the three highest (absolute) correlations involved Alphaproteobacteria SGB4777, positively correlating with the alternate Mediterranean diet (aMED^72^, *ρ* = 0.21) and HEI (*ρ* = 0.19) scores, and negatively correlating with the uPDI (*ρ* = −0.25).

We further compared the SGBs newly linked to diet and biometrics in the ZOE PREDICT 1 re-analysis to those associated with other health and disease conditions in our broader human gut data MetaPhlAn 4 profiles. Among the ten uSGBs most health-associated based on the average correlation ranks with the 19 reference markers selected from the ZOE PREDICT 1 study, *Lachnospiraceae* SGB4894 emerged as a particularly relevant taxon. This uSGB was prevalent in both contemporary human cohorts (44.33% in healthy individuals) and in nonhuman primates (41.36% prevalence). It was also present in 60% of the metagenomes available from ancient stool samples (Supplementary Fig. 8a), suggesting that this taxon is an important, as-yet-uncharacterized member of the healthy human microbiome.

**Fig. 6:**
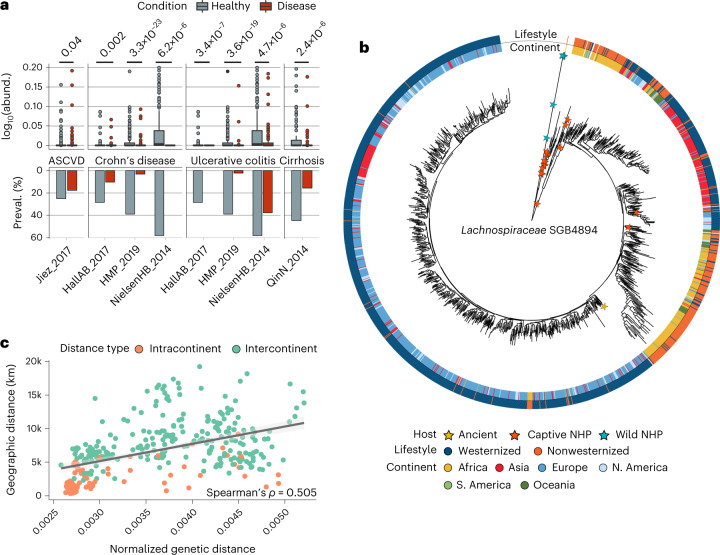
StrainPhlAn 4 accurately reconstructs large-scale strain-level phylogenies of uncharacterized microbial species. **a**, Relative abundances (box plots and top-part *y* axis) and prevalences (bar plots and bottom-part *y* axis) of the uncharacterized species (uSGB) *Lachnospiraceae* SGB4894 are substantially higher in healthy individuals (*n* = 738 samples) in comparison with patients suffering from several gastrointestinal related diseases (*n* = 1,183 samples), and this difference is reproducible across populations (one-sided Mann–Whitney *U* test). Box plots show the median (center), 25th/75th percentile (lower/upper hinges), 1.5× interquartile range (whiskers) and outliers (points). **b**, *Lachnospiraceae* SGB4894 shows within-species genetic diversity strongly linked to geographic origin and lifestyle. **c**, Pairwise geographic distances between strains of different countries correlate with their median genetic distances (Spearman’s *ρ* = 0.505; see Methods), suggesting that human *Lachnospiraceae* SGB4894 strains could have followed an isolation-by-distance pattern.

When comparing the relative abundances of *Lachnospiraceae* SGB4894 in case/control studies across datasets spanning 11 different human diseases (see Methods; Supplementary Table 20), we found statistically significant associations not only with conditions directly linked with cardiometabolic health such as ASCVD (*P* = 0.045) and cirrhosis (*P* = 9.20 × 10^−7^) but also with the inflammatory bowel diseases (IBD; Fig. 6a). This included associations over three different cohorts of a higher abundance and prevalence of *Lachnospiraceae* SGB4894 with both of the main IBD subtypes, Crohn’s disease (*P* = 2.50 × 10^−28^, 4.67 × 10^−6^ and 0.0016) and ulcerative colitis (*P* = 1.85 × 10^−22^, 3.89 × 10^−6^ and 1.28 × 10^−8^). Altogether, these results show the importance of profiling the unknown fraction of the microbiome even for relatively well-characterized environments, such as the human gut, as microbial links with cardiometabolic blood metabolites, dietary patterns and host diseases can also incorporate and shed light on newly defined uSGBs.

### StrainPhlAn 4 reconstructs large phylogenies of uSGBs

The unique clade-specific marker genes exploited by MetaPhlAn to detect and quantify microbial taxa can also be used to reconstruct the sample-specific genetic makeup of individual strains with the StrainPhlAn approach^4,73^. MetaPhlAn 4 also extends StrainPhlAn 4 to be applicable to SGBs, and thus to uncharacterized species (uSGBs). StrainPhlAn 4 uses the MetaPhlAn 4 mapping of reads against markers to produce per-sample genotypes for the dominant strains per species (for all SGBs with sufficient coverage). Compared to StrainPhlAn 3, we improved the procedure to select and process markers and samples with a more robust and validated set of default parameters and a more stringent gap-trimming strategy. We also exploit the larger marker’s database of more phylogenetically consistent SGBs (avg., 189 ± 34 markers per SGB). This resulted in more accurate phylogenies compared to the previous version, with an average of 1.33% increase in correlation between StrainPhlAn phylogenetic distances and MAG-based phylogenies built on the fraction of samples, in which high-quality MAGs could be reconstructed (evaluation done on 100 samples for the three most prevalent kSGBs with consistent MetaPhlAn 3 species; Supplementary Table 21 and Supplementary Fig. 9a–f; see Methods).

To illustrate the potential of StrainPhlAn profiling for uSGBs, we continued our exploration of the health-linked *Lachnospiraceae* SGB4894 introduced above, exploiting the same collection of 19.5 k gut metagenomic samples used for MetaPhlAn 4 (Supplementary Table 12). This analysis incorporated all 5.8 k samples in which MetahlAn 4 detected *Lachnospiraceae* SGB4894, including 79 nonhuman primates and 12 ancient human gut metagenomes (Supplementary Table 22). StrainPhlAn 4 retained 37 SGB4894-specific marker genes (spanning 19,449 nucleotide positions after trimming the alignment to exclude nonvariable positions) across the 1,683 samples, in which the target uSGB had enough coverage for strain profiling (samples with, at least, 20 *Lachnospiraceae* SGB4894 markers reconstructed with >80% breadth of coverage) and automatically built a phylogeny integrating all strain profiles from among host types.

The resulting phylogeny showed that *Lachnospiraceae* SGB4894 is composed of multiple subclades, including one comprising strains mostly from individuals from westernized populations and other two instead dominated by individuals from nonwesternized or Chinese populations, the latter also with higher intraclade diversity (Fig. 6b). One strain reconstructed from a sample of palaeofaeces from ~1,300 years ago^74^ was also integrated within the *Lachnospiraceae* SGB4894 phylogeny and placed as basal for the subclade of mostly European and North American strains (Fig. 6b), whereas the strains from nonhuman primates tended to populate a common, divergent region of the tree.

*Lachnospiraceae* SGB4894’s phylogeny further demonstrated genetic structure linked to the geographic origin of the hosts (Fig. 6b). Indeed, when considering pairs of strains sampled in different countries, we found a correlation between geographic and median genetic distance (Spearman’s *P* = 0.505) that can be used to hypothesize isolation-by-distance effects^75^, as previously shown for *Helicobacter pylori*^76^ and *Eubacterium rectale*^55^ (Fig. 6c). Correspondingly, SGB4894 had a higher intrapopulation genetic variability in nonwesternized populations (Mann–Whitney *U* test, *P* < 2.22 × 10^−46^; Supplementary Fig. 8b) and higher intrasubject polymorphism rates (calculated as the percentage of bases in the reconstructed markers with an allele dominance below 80%, Mann–Whitney *U* test, *P* = 8.6 × 10^-14^; Supplementary Fig. 8c). StrainPhlAn 4 thus readily enabled phylogenetic reconstruction and population genetics for uncultivated, yet-to-be-named species with high precision (see Methods; Supplementary Table 21 and Supplementary Fig. 9g,h).

StrainPhlAn 4 also allows the analysis of strain sharing and transmission between communities^4,25,28,73,77–79^ for uncharacterized species, that is, uSGBs (see Methods). Notably, StrainPhlAn 4 estimated that strains of *Lachnospiraceae* SGB4894 were not shared between mothers and their <1-year infants in all 21 cases in which it was reliably detected in both relatives (Supplementary Fig. 8d). Similarly, only 5.63% of adults in the same household that were positive for *Lachnospiraceae* SGB4894 shared the same strain (Supplementary Fig. 8d), suggesting that stable vertical and horizontal transmission for this species are both rare. There is some evidence for horizontal transmission between host species, however, as we found evidence of two captive nonhuman primates sharing closely related *Lachnospiraceae* SGB4894 strains with humans (Fig. 6b). Overall, this example shows that the extension of StrainPhlAn 4 to incorporate SGBs alongside MetaPhlAn 4 enables the analysis of highly-resolved, sub-species phylogenies for both well-characterized and yet-to-be-cultivated microbial species.

## Discussion

MetaPhlAn 4 provides a strategy for integrating metagenomic assembly with reference-based profiling approaches to achieve novelty by incorporating diverse high-quality metagenome assemblies, and sensitivity and specificity using refined mapping to prescreened marker sequences. This strategy leverages multiple recent large efforts in metagenomically cataloging microbial diversity^37–46^, organizing over 1 M prokaryotic sequences into species-level genome bins, improving the diversity of the microbiome types in comparison to the current biases in available databases and efficiently using them to profile new metagenomes using a marker-based strategy. This approach improved the resolution of health-associated biomarkers and enabled phylogenetic reconstruction and population genetics inference for both known and uncharacterized taxa across tens of thousands of shotgun metagenomes spanning dozens of distinct environments.

Notably, even with the extended MetaPhlAn 4 SGB and marker set, further work remains to better profile under-characterized habitats. Environmental, nonhost-associated, and other under-studied microbial communities are still highly enriched for sequences not captured even by current uSGBs, although the algorithm and software architecture can be continuously updated as new MAGs become available. Indeed, we plan to release at least two new MetaPhlAn databases per year, substantially expanding the profilable microbial diversity. The current methods also do not extensively incorporate viral or eukaryotic microbial sequences, due to their unique genomic architectures and quality control requirements relative to bacterial and archaeal genomes. Interestingly, because SGBs represent essentially whole-genome OTU clusters^80^, many related downstream statistical challenges also remain to be addressed; for example, the tradeoff between sensitivity and specificity when applying quality control measures to identify real but rare taxa. Another important aspect of increasing relevance in current metagenomic research is the phylogenetic and taxonomic contextualization of under-characterized species, specifically uSGBs. While MetaPhlAn 4 has been designed to provide taxonomic labels corresponding to the part of the taxonomy that can be confidentially transferred from the closest (if any) reference genomes, and PhyloPhlAn^81^ provides specific workflows for phylogenetic characterization, further integration of isolate genomes and new methods for defining taxonomic clades above the level of the microbial family are still needed. We expect to continue addressing these challenges in future versions of the methodology, which will also form the basis for other MAG-aware updates of the bioBakery platform^4,82^.

## Methods

### Overview of the approach

MetaPhlAn 4 taxonomic profiling relies on detecting the presence and estimating the coverage of a collection of species-specific marker genes to estimate the relative abundance of known and unknown microbial taxa in shotgun metagenomic samples. Since version 4, MetaPhlAn is relying on the concept of sequence-defined species-level genome bins (SGBs)^42^ that addresses many limitations of manual taxonomy assignment and encompasses taxonomic units both with available reference genomes from cultivation (kSGBs) and taxa defined solely based on the metagenome-assembled genomes (uSGBs).

As a brief summary of the approach (details in the following subsections), to build the MetaPhlAn database of SGB-specific markers, we collected a catalog of 729,195 dereplicated and quality-controlled genomes (560,084 MAGs and 169,111 reference genomes) that was used to expand the SGB organization by Pasolli et al. ^42^. This led to the definition of 21,373 FGBs, 47,643 genus-level genome bins (GGBs) and 70,927 SGBs, with 23,737 of them containing at least one reference genome (kSGBs) and 47,190 containing only MAGs (uSGBs). To minimize the chance that SGBs incorporate assembly artifacts or chimeric sequences, we considered only those uSGBs with at least five MAGs (no filtering for kSGBs). The genome catalog was then annotated using the UniRef90 database^58^ (see below) and, within each SGB, the genes that could not be assigned to UniRef90 gene families were de novo clustered together using the UniClust90 (ref. ^59^) criteria (>90% identity and >80% coverage of the cluster centroid). Using the resulting UniRef- and UniClust90 annotations, we defined a set of core genes for each quality-controlled SGB (genes present in almost all genomes composing an SGB), and after mapping all core genes against the entire genomic catalog, we defined a set of 5.1 M SGB-specific marker genes (core genes not present in any other SGB) for a total of 21,978 kSGBs and 4,992 uSGBs.

For the taxonomic profiling step that uses the markers based on the SGB data, MetaPhlAn 4 maps metagenomic reads (preferably already quality controlled) against the marker database using Bowtie 2 (ref. ^83^). From these mapping results, MetaPhlAn estimates the coverage of each marker and computes the clade’s coverage as the robust average of the coverage across the markers of the same clade. Finally, the clade’s coverages are normalized across all detected clades to obtain the relative abundance of each taxon. Several downstream analyses are included in the MetaPhlAn package, including the strain-level phylogenetic profiling of SGBs by StrainPhlAn.

### The starting catalog of reference genomes and MAGs

Starting from the original catalog of 154,724 human MAGs and 80,990 reference genomes collected by Pasolli et al. ^42^, we retrieved an additional set of 616,805 MAGs spanning different human body sites, animal hosts and nonhost-associated environments (Supplementary Table 2), and 155,767 new reference genomes available as of November 2020 in the NCBI Genbank database^84^. To ensure the quality of the downloaded sequences, we executed CheckM version 1.1.4 (ref. ^85^) on the complete catalog of 1,008,148 genomes (that is, reference sequences and MAGs), filtering those with completeness below 50% or contamination above 5%. To avoid multiple inclusions of the same strains, we computed the all-versus-all MASH distances^54^ (version 2.0) on the quality-controlled sequences, followed by the dereplication at 99,99% genetic identity. This resulted in a quality-controlled catalog of 729,195 genomes, comprising 560,084 MAGs and 169,111 reference genomes.

### Building the expanded SGB catalog

Using the new genomic catalog, we expanded the SGB organization proposed by Pasolli et al. ^42^. First, we apply the ‘*phylophlan_metagenomic’* subroutine of PhyloPhlAn 3 (ref. ^81^) on the 493,482 new MAGs and reference genomes to identify their closest SGB, GGB and FGB and their MASH distances. Based on the reported distances, we assigned the genomes to the already existing SGBs, GGBs and FGBs according to the thresholds defined by Pasolli et al. (5%, 15% and 30% genetic distance, respectively)^42^. We then applied a hierarchical clustering with average linkage on the all-versus-all MASH distances of the genomes not assigned to any existing SGB, using the ‘fastcluster’ python package version 1.1.25. The resulting dendrogram was divided with cutoffs at 5%, 15% and 30% genetic distance to define 54,596 new SGBs, 37,546 new GGBs and 18,211 new FGBs, respectively. In short, from the initial filtered catalog of 729,195 MAGs and reference genomes, we defined 21,373 FGBs, 47,643 GGBs and 70,927 SGBs, with 23,737 of them containing, at least, one reference genome (kSGBs) and 47,190 containing only MAGs (uSGBs; Supplementary Table 1). In comparison with the latest largest MAG collections^43,45^, our genome catalog spans 5,092 more kSGBs and 19,121 more uSGBs.

We assigned a taxonomic label to all 70,927 SGBs according to the NCBI taxonomy database (as of February 2021)^49^. For kSGBs, we assigned taxonomy by applying a majority rule to the taxonomic labels of the reference genomes contained in each SGB. In case of a tie, the taxonomic label is resolved by choosing the representative taxon, the one alphabetically first. For uSGBs, we applied a similar majority rule but on the taxonomies of the reference genomes contained at the GGB level, assigning a taxonomic label up to the genus level. If no reference genomes were present at the GGB level, we further applied the same procedure at the FGB level. If no reference genomes were found at the FGB level, we assigned the taxonomic labels only up to the phylum level by considering the phylum that is most recurrent within the set of taxonomic labels of the closest reference genome and, at most, up to one hundred reference genomes within 5% genomic distance to the closest as identified by ‘*phylophlan_metagenomic*’. For the taxonomic levels not receiving any taxonomy label, we assigned all the internal taxonomic nodes with SGB, GGB and FGB identifiers to maintain the taxonomy with all its levels and for providing categorization of uSGBs.

### Genome annotation and pangenome generation

The filtered catalog of 729,195 MAGs and reference genomes was subjected to an annotation workflow, in which (1) the FASTA files were processed with Prokka (version 1.14)^86^ to detect and annotate the coding sequences (CDS) and (2) subsequently assign the CDS to a UniRef90 cluster^58^ using a DIAMOND-based pipeline (available in https://github.com/biobakery/uniref_annotator). The DIAMOND-based pipeline performs a sequence search (DIAMOND version 0.9.24)^87^ of the protein sequences against the UniRef90 database (release 2019_06) and then applies the UniRef90 inclusion criteria on the mapping results to annotate the input sequences (>90% identity and >80% coverage of the cluster centroid). Within each SGB, protein sequences that were not assigned to any UniRef90 cluster were clustered using MMseqs2 (ref. ^88^) following the Uniclust90 criteria (‘*-c 0.80–min-seq-id 0.9*’ parameters)^59^.

For each SGB, based on the UniRef90 and UniClust90 annotations, a pangenome was generated by collecting all the UniRef/UniClust90 clusters present in at least one of the SGB’s genomes. For each cluster, the representative sequence was randomly selected within all the genomes and a coreness value was calculated based on the cluster prevalence within the 2 k highest quality genomes of the SGB. uSGBs containing less than five MAGs were discarded for the following steps. We implemented this restriction because we found evidence that some of the small uSGBs contained likely assembly artifacts or chimeric genomes, and they were also more likely to generate false positives by failing to omit potential markers that later proved to be ambiguous. In this step, 41,498 uSGBs of the 70,927 SGBs were discarded, while all kSGBs were retained as they are represented by theoretically more reliable sequences.

### The MetaPhlAn 4 vJan21 markers database

From these pangenomes, the construction of the marker database for MetaPhlAn 4 is divided into two sequential steps as follows: the identification of the core genes within each SGB and the screening of the core genes for their SGB-specificity.

For the identification of the core genes, the procedure first defines a coreness percentage threshold (that is, the percentual prevalence of a gene within the SGB) based on the SGB pangenomes. Specifically, we selected the maximum coreness threshold that allowed the retrieval of at least 800 core genes (of length between 450 and 4,500 nucleotides). The minimum coreness threshold was bound to 60% for SGBs with less than 100 genomes and 50% for the others. For each SGB, a core gene set was generated using the inferred coreness thresholds. On average, we retrieved 2,985 core genes per SGB (median, 2,687; s.d., 1,861). SGBs with less than 200 core genes were discarded and not considered further (9 SGBs).

To detect the SGB-specific marker genes, each set of core genes was then aligned against the genomes of the other SGBs using Bowtie 2 (version 2.3.5.1; --*sensitive* parameter)^83^. For each SGB, a subset comprising up to the highest quality 100 genomes was selected for the mapping for computational reasons. Each core gene was split into fragments of 150-nt length to simulate metagenomic reads, and then they were mapped against the representative subset of the SGB’s genomes. An alignment hit of a fragment was considered a hit for its corresponding core gene. Core genes hitting none (perfectly unique markers) or less than 1% (quasi-markers) of the genomes of any other SGB and hitting a number of the genomes of their SGB above or equal to their coreness threshold were selected as marker genes. Crucially, this uniqueness procedure was substantially stricter than those used in previous MetaPhlAn versions owing to the improved consistency of the SGBs compared to original species taxonomic assignments.

The small fraction of SGBs producing less than 100 marker genes (810 SGBs) was subjected to the following workflow:

1. If more than 200 core genes of the target SGB were matching an external SGB (a kSGB belonging to the same species, or a uSGB) and if the external SGB had less than 10% of the genomes in the target SGB, then the external SGB was discarded (this occurred for 392 kSGBs and 150 uSGBs). This step was repeated every time an external SGB was removed until the target SGB produced 100 marker genes or there were no more external SGBs that could be evaluated. In the latter case, the removal of the external SGBs was rolled back.
2. If the target SGB still could not identify ten marker genes, external SGBs with low-quality species taxonomic labels were discarded (this occurred for 822 kSGBs and 286 uSGBs). Specifically, the regular expressions used to detecting low-quality species taxonomic labels are
  ‘(C|c)andidat(e|us) | _sp(_.*|$) | (.*_|^)(b|B)acterium(_.*|) |.*(eury|)archaeo(n_|te|n$).* |.*(endo|)symbiont.* |.*genomosp_.* |.*unidentified.* |.*_bacteria_.* |.*_taxon_.* |.*_et_al_.* |.*_and_.* |.*(cyano|proteo|actino)bacterium_.*) This step was repeated every time an external SGB was removed until the target SGB produced ten marker genes or there were no more external SGBs that could be evaluated. In the latter case, the removal of the external SGBs was rolled back.
3. For the SGBs that still did not produce at least ten marker genes, a conflict graph was generated collecting all the core gene hits against external SGBs in which more than 200 core genes were in conflict. The graph was then processed by merging SGBs with a procedure that minimizes the number of merged SGBs and maximizes the number of markers retrieved. After this process, 849 SGBs were merged, producing 237 SGB groups.

Finally, for each SGB, a maximum of 200 marker genes were selected based first on their uniqueness and then on their size (longer first). SGBs that still had fewer than ten markers were discarded (188 SGBs). Each marker was associated with an entry in the MetaPhlAn 4 vJan21 database which includes the SGB for which the sequence is a marker, the list of SGBs sharing the marker, the sequence length, and the taxonomy of the SGB. This produced a list of 5.1 M marker genes for a total of 21,978 kSGBs and 4,992 uSGBs (4,863 kSGBs and 1,198 uSGBs still not captured by the latest largest genomes catalogs^43,45^).

### MetaPhlAn 4 taxonomic profiling

MetaPhlAn 4 taxonomic profiling is based on the read homology to and coverage of SGB-specific markers to estimate the relative abundance of taxonomic clades present in a metagenomic sample. The MetaPhlAn pipeline starts by mapping the raw reads of metagenomic samples against the SGB-specific markers’ database using Bowtie 2 (ref. ^83^). Input metagenomic reads can be provided as a single FASTQ file (compressed with several algorithms), multiple FASTQ files included in a single (compressed) archive, or as a preperformed mapping (*bowtie2out* format). By default, the Bowtie 2 mapping is performed using the ‘--*very-sensitive*’ preset. For read-mapping quality purposes, short reads (reads shorter than 70 bp; ‘*--read_min_len*’ parameter) and low-quality alignments (alignments with a MAPQ value lower than 5; ‘*--min_mapq_val*’ parameter) are discarded.

Using the quality-controlled mapping results, MetaPhlAn estimates the coverage of each marker and computes the clade’s coverage as the robust average of the coverage across the markers of the same clade, but excluding the top and bottom quantiles of the marker abundances (‘*--stat_q*’ parameter). For the SGB profiling, this parameter by default set to 0.2, thus excluding the 20% of markers with the highest abundance and the 20% of markers with the lowest abundance. The coverage of quasi-markers is not considered from this computation when at least 33% (default value, ‘*--perc_nonzero*’ parameter) of the markers of their respective external SGB were present. The clade’s coverages are finally normalized across all detected clades to obtain the relative abundance of each taxon as previously described in (refs. ^2,3^).

### MetaPhlAn 4 compatibility with the GTDB taxonomy

MetaPhlAn 4 supports additional taxonomies via genome and MAG matching against other systems. We specifically implemented the mapping of the MetaPhlAn 4 SGB-based taxonomic profiles to those based on the species in the GTDB^63^. This is available via the utility script ‘*sgb_to_gtdb_profile.py*’ included in the version 4 release. To assign each SGB to a GTDB species, we used the GTDB-Tk taxonomic classification tool (version 2.1.1)^89^ to assign a GTDB-defined species (release 207) to each centroid genome of the 26,970 SGBs included in the MetaPhlAn 4 database.

### MetaPhlAn 4 unclassified reads calculation

MetaPhlAn 4 includes a feature for estimating the fraction of input reads that cannot be assigned to taxa in the database (‘*--unclassified_estimation*’ parameter). This is calculated by subtracting from the total number of input reads the average read depth of each reported SGB normalized by its SGB-specific average genome length as follows:

$$\begin{array}{l}\% {\mathrm{uncl.}\mathrm{reads}} =\\ \frac{{{\mathrm{Total}\,\mathrm{reads}} - \left( {\mathop {\sum}\nolimits_{{\mathrm{sp}} = 0}^{n} {\left( {{\mathrm{avg}\,\mathrm{non} \mathrm{zero}\,\mathrm{markers}\,\mathrm{coverage}_\mathrm{sp} \times {\mathrm{avg}\,\mathrm{genome}\,\mathrm{length}_{\mathrm{sp}}}}} \right)} } \right) /{\mathrm{avg}\,\mathrm{read}\,\mathrm{length}}}}{{{\mathrm{Total}\,\mathrm{reads}}}}\end{array}$$

$${\mathrm{sp} = \mathrm{indices}\,\mathrm{of}\,\mathrm{all}\,\mathrm{the}\,\mathrm{SGBs}\,\mathrm{reported}\,\mathrm{in}\,\mathrm{the}\,\mathrm{MetaPhlAn}\,\mathrm{profile}}$$

The average read depth of a SGB is calculated as the mean read depth of all its detected (nonzero) marker genes. The SGB-specific genome length for kSGBs is calculated using only the genome lengths of its reference genomes, while for uSGBs the average genome length is incremented by 7% (calculated to be the average difference between the genome sizes of references genomes and MAGs within the same SGB).

### Building the MetaPhlAn 4 tree of life

The MetaPhlAn 4 package includes the phylogenetic tree of all the SGBs available in the MetaPhlAn database (the ‘microbial tree of life’; Supplementary Fig. 10), enabling both the calculation of phylogeny-based beta-diversity estimates between samples such as the UniFrac^90^ (Supplementary Fig. 4), and the further exploration of phylogenetic relations between SGBs. To build the tree, we selected the highest quality genomes for each of the 26,970 SGBs based on the CheckM. We then executed PhyloPhlAn 3 (ref. ^81^) with the optimized set of parameters for very large phylogenies as described in (ref. ^81^). In particular, PhyloPhlAn performed a DIAMOND^87^ mapping (version 0.9.24) against the 400 PhyloPhlAn’s universal markers’ database, used TrimAl version 1.4.rev15 (ref. ^91^) for the trimming, MAFFT version 7.475 (ref. ^92^) to generate the multiple-sequence alignment, and IQ-TREE version 2.0.3 (ref. ^93^) for the phylogenetic reconstruction, together with the PhyloPhlAn presets ‘--*diversity high–fast*’.

### MetaPhlAn 4 synthetic evaluation

We evaluated MetaPhlAn 4 using different published and newly created synthetic metagenomes. Firstly, we assessed the performance of MetaPhlAn 4 in comparison to several available alternatives, that is, MetaPhlAn 3 (ref. ^4^), mOTUs 2.6 (latest database available as of March 2021)^6^ and Bracken 2.5 (ref. ^5^). Through the OPAL benchmarking framework^61^, we evaluated the performance of each tool by profiling the CAMI 2 taxonomic profiling challenge metagenomes^60^ and SynPhlAn-nonhuman synthetic metagenomes^4^. The CAMI 2 metagenomes include 128 samples representing five human body site-specific microbiomes (that is, airways, oral, the gastrointestinal tract, skin and the urogenital tract), the marine environment and the mouse gut microbiome, while the SynPhlAn-nonhuman metagenomes were designed to mirror the sequencing depth and community structure of the CAMI 2 metagenomes (that is, 30 million, 150-nt paired-end sequencing reads from genomes in kSGBs with a log-normal abundance distribution), but for environments different than the human body.

We ran each tool using default parameters. For mOTUs 2.6, we considered two different settings, and it was thus run twice with parameters ‘*-C recall*’ and ‘*-C precision*’ to optimize for precision and recall separately, respectively. Both parameters are preset configurations of mOTUs 2 created by its developers for the CAMI 2 challenge. Results from Bracken 2.5 were filtered out discarding species reported with a relative abundance below 0.01%. Additionally, to better evaluate the SGB architecture, we performed an alternative evaluation assessing the detection and quantification of the genomes included in the synthetic metagenomes. To this end, we defined (1) ‘true positive’ as the detection of an SGB containing a genome present in the synthetic metagenome, (2) ‘false positive’ as the detection of an SGB that does not contain any genome in the metagenome and (3) ‘false negative’ as the nondetection of an SGB containing a genome present in the synthetic metagenome. Detection of an SGB that represents an overlapping SGB present in the community was also accounted as ‘true positive’. For the gold standard, relative abundances were obtained by summing up the relative abundances of the genomes belonging to the same SGB. For MetaPhlAn 3 that contains markers describing species groups, we considered (1) ‘true positive’ a species group containing a species present in the synthetic metagenome and (2) ‘false positive’ a species group that does not contain any species present in the synthetic metagenome.

To further assess the performance of MetaPhlAn 4 to profile both known and unknown SGBs complementing the synthetic samples from CAMI 2 and SynPhlAn, we constructed additional synthetic metagenomes from different environments, hosts and human body sites using ART^94^ with the Illumina HiSeq 2500 error model (available at http://segatalab.cibio.unitn.it/tools/metaphlan/). For each environment, we simulated five metagenomes containing 30 million, 150-nt paired-end sequencing reads using randomly selected genomes from SGBs containing MAGs coming from that environment (with a restriction of one genome per SGB), and following a log-normal abundance distribution. MetaPhlAn 4 evaluation was then performed by assessing the detection and quantification of the genomes included in the synthetic metagenomes as described above. Additionally, to demonstrate that the evaluation was not biased by the usage of genomes included in the genomic catalog, we built, using the same procedure, another five metagenomes using a mixture of new MAGs and reference genomes not included in our genomic database. SGB assignment of the new genomes was performed using the ‘*phylophlan_metagenomic’* subroutine of PhyloPhlAn 3 (ref. ^81^) against the Jan21 database.

Finally, to assess the minimum relative abundance at which MetaPhlAn 4 can confidently assign a species, we randomly selected five reference genomes and five MAGs from the mixture genomes not included in our genomic database to simulate single-isolate synthetic metagenomes at different depths of coverage using ART^94^ with the Illumina HiSeq 2,500 error model (available at http://segatalab.cibio.unitn.it/tools/metaphlan/). For each genome, we generated reads at 0.01×, 0.05×, 0.1×, 0.5×, 1×, 5×, 10×, 50× and 100× coverage.

### MetaPhlAn 4 application to human and nonhuman metagenomes

To measure the increase of the fraction of classified reads when compared with MetaPhlAn 3, we profiled 24,515 samples from 145 datasets spanning different human body sites (airways, gastrointestinal tract, oral, skin and urogenital tract) and lifestyles, animal hosts (nonhuman primates, mice and ruminants) and other nonhost-associated environments (soil, fresh water and ocean) (Supplementary Table 11) with both MetaPhlAn 3 (version 3.0.12) and MetaPhlAn 4 (version 4.beta.1) using the unknown/unclassified estimation feature (Supplementary Table 23). Improvements were reported using only the samples in which both tools reported, at least, one species. An SGB was reported to be present in a specific environment if it was detected in, at least, 1% of the samples from that environment. Finally, to investigate the abundance and prevalence of gut-related SGBs across different age categories and lifestyles, we selected a subset of 19,468 human gut metagenomes from 86 datasets for which age information was available (Supplementary Table 15) as reported and curated in the curatedMetagenomicData^95^ 3 package.

### Westernization definition

The process of westernization brought by industrialization and urbanization over the past two hundred years has had a significant impact on human populations. These changes include access to pharmaceuticals and healthcare, improved sanitation and hygiene, increased urban dwelling and decreased exposure to livestock; and changes in habitual diets (with westernized diets tending to consist of increased fat and animal proteins, high salt and simple carbohydrates). In this study, we characterize westernized or nonwesternised individuals or populations based on either the distinction given in the primary publication or an assessment based on the criteria outlined above.

### Analysis of diet-related taxa in the mouse microbiome

We performed a differential abundance analysis of HF versus normal chow diets in a public cohort of 181 mouse gut microbiome^65^. From the original cohort, we excluded ten samples missing age information, and we selected only the samples from genetic backgrounds tested for both types of diet. In total, we analyzed 43 HF-fed mice and 88 mice fed with normal control chow, further stratified into two genetic backgrounds and five vendors (Supplementary Table 17). To correct data compositionality, we first imputed the zero values with the minimum value for an abundance found in the dataset, then we applied the centered-log-ratio transformation to the SGB’s relative abundance distribution (‘*scikit-bio*’ Python package, version 0.5.6). We then built a random-intercept model for each feature (SGB) using the ‘*statsmodels*’ Python package version 0.11.1. We associated the diet (HF or chow, encoded as a binary factor) to the transformed abundance of the strain, using the sex, age-in-days and genetic background of the mice as fixed effects and the vendor as a grouping variable. Significance was determined by the Wald test. *P* values were corrected according to Benjamini-Hochberg (‘*statsmodels*’ Python package, *Q* < 0.2). Before plotting, we selected the biomarkers having a mean abundance in the associated group greater than 1%. The reported heatmap was printed using the ‘*pheatmap*’ R package version 1.0.12 (parameters ‘*clustering_distance_cols* = *‘euclidean’, clustering_method* = *‘complete’, cluster_rows* = *FALSE*’).

### Re-analysis of the ZOE PREDICT 1 intervention study

We assessed the associations between microbiome and cardiometabolic health and dietary patterns using 1,001 deeply phenotyped individuals from the United Kingdom retrieved from the ZOE PREDICT 1 intervention study^22^. Machine learning (ML) analyses were performed using the ‘*scikit-learn*’ Python package (version 0.22.2) on a panel of 19 representative nutritional and cardiometabolic markers described in the original study^22^. A cross-validation approach was implemented with a random split of 80/20 of training and testing sets, repeated for 100 bootstrap iterations, again with the same exact approach as the original study. Because the ZOE PREDICT 1 cohort includes twins, to avoid overfitting, the twin from the training set was removed if its twin pair was present in the testing set. The ML model is based on RFs using SGBs-level taxonomic relative abundances as estimated by MetaPhlAn 4 and relative abundance values were arcsin-sqrt transformed.

For the RF classification task, continuous features were divided into two classes, the top and bottom quartiles. The ‘*RandomForestClassifier’* function was used with parameters ‘*n_estimators*=*1000, max_features*=*‘sqrt’*’. For the RF regression task, the RandomForestRegressor function was used with parameters ‘*n_estimators*=*1000, criterion*=*‘mse’, max_features* = *‘sqrt’*’. A linear regressor (‘*LinearRegression’* function with default parameters) was also trained on training target values to calibrate the range of output values predicted by the RF regressor model. Pairwise Spearman’s correlations were calculated between the relative abundance of uSGBs with a prevalence of at least 20% (at least 200 of 1001 samples), and the panel of 19 nutritional and cardiometabolic markers, correcting for age, sex and body mass index. Correlations were computed using the ‘*ppcor*’ R package version 1.1 (Supplementary Table 24) and *P* values were corrected through the Benjamini-Hochberg procedure.

### *Lachnospiraceae* SGB4894 association with health conditions

To investigate associations between *Lachnospiraceae* SGB4894 with host health conditions across several diseases, we collected 21 disease case–control datasets available through curatedMetagenomicData^95^ (Supplementary Table 20). For each dataset, we assessed the associations of *Lachnospiraceae* SGB4894 with the subjects reported as healthy controls by computing a one-sided Mann–Whitney *U* test on the arcsin square root transformed relative abundances profiles using the ‘*stats.mannwhitneyu*’ function of the ‘*scipy*’ Python package version 1.5.2. Samples from westernized adults were used and comparisons were performed only when at least ten healthy and ten disease samples were available. Statistically significant associations were defined by a *P* < 0.05.

### StrainPhlAn 4 profiling

StrainPhlAn profiling estimates strain-level species-specific phylogenies, and it is based on the reconstruction of sample-specific consensus sequences of MetaPhlAn species-specific marker genes followed by multiple-sequence alignment and phylogenetic inference^4,73^. Compared to StrainPhlAn 3, the accuracy and performance of StrainPhlAn 4 have been improved mostly because of (1) the redesigned procedure to select and process markers and samples to be considered in the phylogeny, and (2) the use of the same MetaPhlAn 4 database of markers from the extensive set of phylogenetically consistent SGBs.

For item (1), StrainPhlAn 4 considers as input the reads-to-markers alignment results (in SAM format^96^) from the MetaPhlAn 4 profiling together with the MetaPhlAn 4 database. For each sample, StrainPhlAn 4 reconstructs consensus sequences of the species-specific marker genes by considering, for each position, the nucleotide with the highest frequency among the reads mapping against it. By default, consensus markers covered by less than eight reads or with a breadth of coverage below 80% are discarded (that is, the proportion of the marker covered by reads, ‘*--breadth_threshold*’ parameter). For this step, ambiguous bases (that is, positions in alignment with quality lower than 30 or with major allele dominance below 80%) are considered unmapped positions. After the reconstruction of the markers, StrainPhlAn discards samples with less than 80% of the available markers and markers present in less than 80% of the samples (‘*--sample_with_n_markers*’ and ‘*–marker_in_n_samples*’ parameters, respectively). Then, markers are trimmed by removing the leading and trailing 50 bases (‘*–trim_sequences*’ parameter), and a polymorphic rates report is generated. Finally, the remaining samples and markers are processed by PhyloPhlAn^81^. By default, multiple-sequence alignment is performed by MAFFT^92^, gappy positions (that is, positions with more than 67% of gaps) are trimmed by trimAl^91^ and phylogenetic trees are inferred by RAxML^97^.

### *Lachnospiraceae* SGB4894 strain-level analyses

For the *Lachnospiraceae* SGB4894 strain-level analysis, we selected 5,883 human gut metagenomic samples from 86 datasets in which *Lachnospiraceae* SGB4894 was reported to be present based by MetaPhlAn 4 (Supplementary Table 15). Seventy-nine nonhuman primates (NHP) and 12 ancient human gut metagenomic samples were also included from 12 different datasets (Supplementary Table 22). SGB4894-specific marker genes were successfully reconstructed from 2,787 metagenomes, of which 2,738 were from contemporary human gut microbiome samples, five from ancient gut microbiome samples, and 44 from NHP gut microbiome samples. Strain-level profiling with StrainPhlAn 4 was performed using parameters ‘*--marker_in_n_samples 70 —sample_with_n_markers 10 ––phylophlan_mode accurate*’. The phylogenetic tree generated by StrainPhlAn was plotted with GraPhlAn version 1.1.4 (ref. ^98^). Phylogenetic distances were extracted based on the distance between samples in the tree and normalized by the total branch length of the tree. Geographic distances between countries were calculated using the ‘*distGeo*’ function of the ‘*geosphere*’ R package version 1.5–10. Spearman’s correlation between genetic and geographic distance was then calculated using the ‘*cor.test*’ function of the ‘*stats’* R package version 4.0.5. Finally, to assess the transmissibility of *Lachnospiraceae* SGB4894, we executed the StrainPhlAn’s ‘*strain_transmission.py*’ script using as input the phylogenetic tree (default parameters). The script, which is part of the StrainPhlAn release, can use the species-specific cutoffs on the normalized phylogenetic distances precomputed on the available datasets with longitudinal sampling.

### StrainPhlAn 4 evaluation

The three most prevalent single-species kSGBs whose species were available in the MetaPhlAn 3 database, that is, *B. wexlerae* (SGB4837), *B. uniformis* (SGB1836) and *E. rectale* (SGB4933), were selected to evaluate the improvements included in StrainPhlAn 4 in comparison with the previous version. As a gold standard, for each species, we considered 100 high-quality MAGs randomly selected from the genomic catalog (Supplementary Table 25) and obtained a phylogeny by processing the MAGs via Roary core gene alignment and RAxML tree reconstruction. Specifically, we computed a multiple-sequence alignment from each set of core genes (present in at least 90% of genomes) using Roary version 3.13.0 (ref. ^99^) with parameters ‘*-cd 90 -i 90 -e --mafft*’, and launched RAxML version 8.2.4 (ref. ^97^) with parameters ‘*-f a -# 100 -p 12345 -x 12345 -m GTRGAMMA*’. Using the metagenomic samples from which the considered MAGs were assembled, we executed StrainPhlAn 3 and 4 using their respective database and with default parameters and ‘*–mutation_rates*’. Additionally, we executed a similar evaluation (but using the MetaPhlAn 4 database in the StrainPhlAn 3 call) on the uSGB *Lachnospiraceae* SGB4894, using the 170 MAGs from the genomic catalog with publicly available metagenomic samples. Pairwise phylogenetic distances normalized by the total branch length were calculated using the PyPhlAn package (https://github.com/SegataLab/pyphlan). Pearson correlations between StrainPhlAn and the gold standard results were calculated using the ‘*stats.pearsonr*’ function of the ‘*scipy*’ Python package version 1.5.2.

### Reporting summary

Further information on research design is available in the Nature Portfolio Reporting Summary linked to this article.

## Online content

Any methods, additional references, Nature Portfolio reporting summaries, source data, extended data, supplementary information, acknowledgements, peer review information; details of author contributions and competing interests; and statements of data and code availability are available at 10.1038/s41587-023-01688-w.

### Supplementary information

Supplementary Information Supplementary Figs. 1–10 and Supplementary Tables 1–25.

Reporting Summary

Supplementary Tables Supplementary Tables 1–25.

Supplementary Table 2 Zip file of Supplementary Table 2.

## Data Availability

All metagenomic studies analyzed in this work are publicly available through the corresponding publications listed in Supplementary Table 11. All reference genomes and taxonomic data are publicly available through the NCBI GenBank database (https://www.ncbi.nlm.nih.gov/genbank/). The GTDB release 207 is publicly available at https://gtdb.ecogenomic.org/. The CAMI 2 Challenge synthetic metagenomes and gold standards are available at https://www.microbiome-cosi.org/cami/cami/cami2. The SynPhlAn-nonhuman synthetic metagenomes and gold standards are available at http://segatalab.cibio.unitn.it/tools/biobakery. The new synthetic metagenomes containing kSGBs and uSGBs and gold standards as well as the single-isolate synthetic metagenomes are available at http://segatalab.cibio.unitn.it/tools/metaphlan/. Prevalences of the SGBs across environments, age categories and lifestyles are available in Supplementary Tables 13 and 14. Metadata of the publicly analyzed human metagenomes is also available through the curatedMetagenomicData R package^95^. The full list of metagenomic studies used for the strain-level analysis of *Lachnospiraceae* SGB4894 is reported in Supplementary Tables 15 and 22.

## Code availability

The MetaPhlAn 4 version described in this work is labeled as MetaPhlAn 4.beta.1 and is available at http://segatalab.cibio.unitn.it/tools/metaphlan with the open source code at https://github.com/biobakery/MetaPhlAn (ref. ^100^) together with StrainPhlAn 4. It is also available via Bioconda https://anaconda.org/bioconda/metaphlan (ref. ^101^) and PIP https://pypi.org/project/MetaPhlAn.
