# Supplementary material for: Extending and improving metagenomic taxonomic profiling with uncharacterized species using MetaPhlAn 4
Source: Nat Biotechnol. 2023 Feb 23;41(11):1633–44. doi: 10.1038/s41587-023-01688-w (PMC10635831; doi:10.1038/s41587-023-01688-w)
Supplement: Supplementary file 1 — Supplementary Figs. 1–10 and Supplementary Tables 1–25. [file 41587_2023_1688_MOESM1_ESM.pdf]

nature biotechnology

Article

https://doi.org/10.1038/s41587-023-01688-w

Extending and improving metagenomic taxonomic profiling with uncharacterized species using MetaPhlAn 4

In the format provided by the authors and unedited

Supplementary Figures

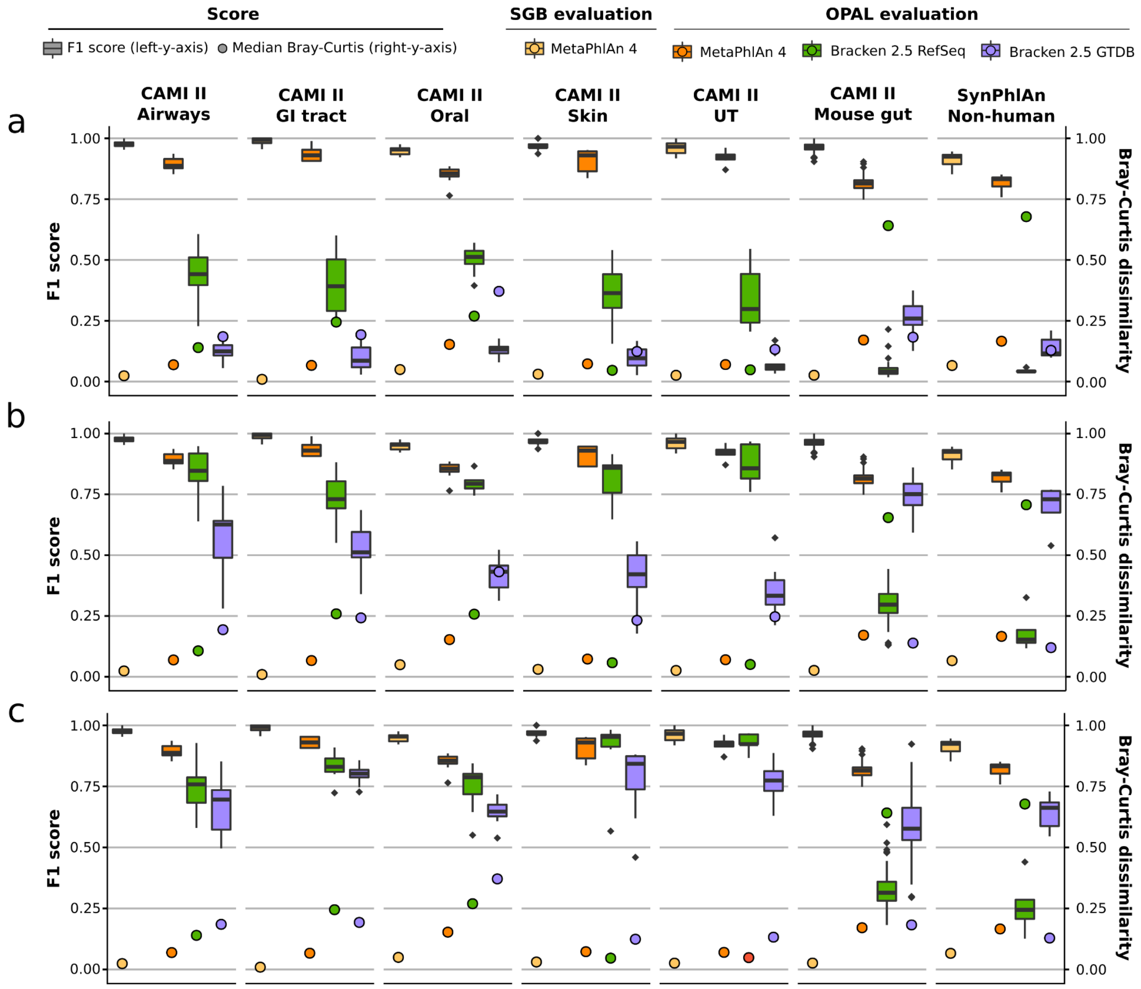

Supplementary Figure 1: Performance of Bracken 2.5 using different minimum relative abundance thresholds. Evaluation (n=123 samples) using (**a**) no threshold, (**b**) 0.01% threshold, and (**c**) 0.1% threshold. Box plots in **a** and **b** show the median (center), 25th/75th percentile (lower/upper hinges), 1.5× interquartile range (whiskers) and outliers (points).

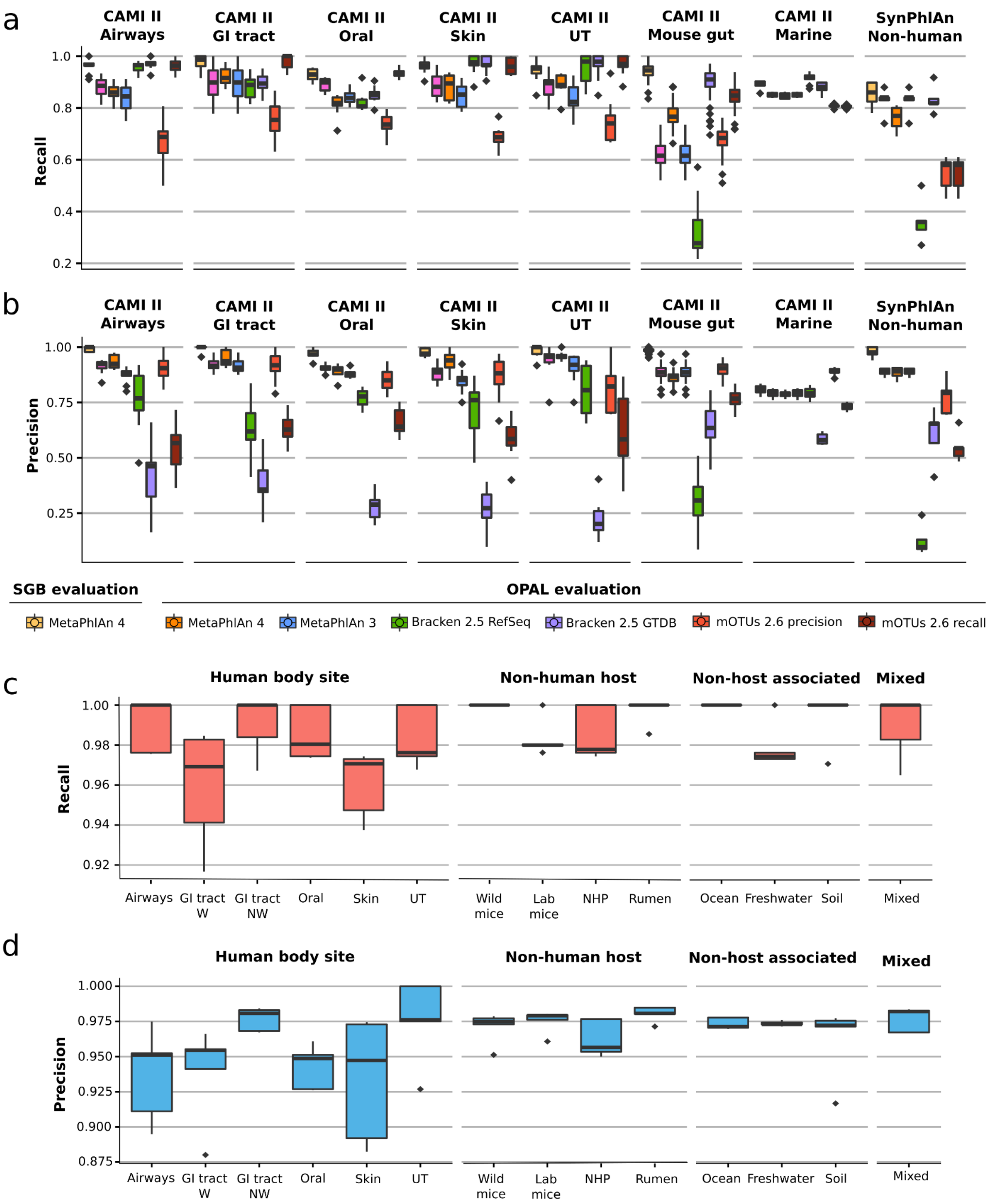

Supplementary Figure 2: Performance of MetaPhlAn 4 in comparison with several available alternatives. a-b, Evaluation using the CAMI II taxonomic profiling challenge (n=128 samples) and SynPhlAn-nonhuman synthetic metagenomes (n=5 samples). **c-d,** Evaluation using the new synthetic dataset containing both known and unknown SGBs (n=70 samples). MetaPhlAn 4 shows high accuracy when assessing (**a**,**c**) recall and (**b**,**d**) precision. GI=gastrointestinal, UT=urogenital tract. Box plots in **a**, **b**, **c** and **d** show the median (center), 25th/75th percentile (lower/upper hinges), 1.5× interquartile range (whiskers) and outliers (points).

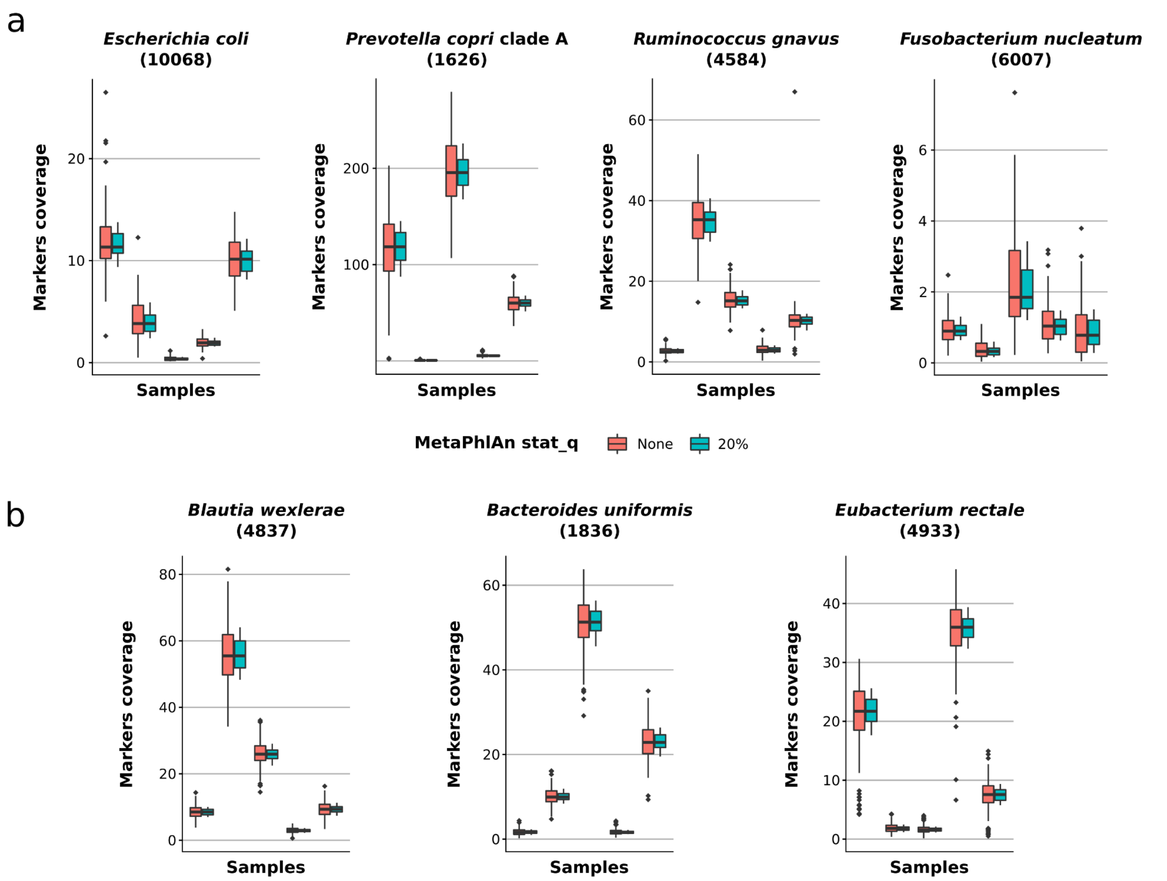

Supplementary Figure 3: Per sample coverage of the MetaPhlAn 4 markers. MetaPhlAn 4 markers show a high coverage consistency when assessing (**a**) biologically interesting species (n=5 samples per SGB) as well as (**b**) the three most prevalent kSGBs (n=5 samples per SGB). Top y-axis (red boxplots) represents the markers' coverage without applying any stat_q filtering and the bottom y-axis (blue boxplots) represents the markers' coverage while applying MetaPhlAn 4 default stat_q filtering (20%, see **Methods**). Box plots in **a** and **b** show the median (center), 25th/75th percentile (lower/upper hinges), 1.5× interquartile range (whiskers) and outliers (points).

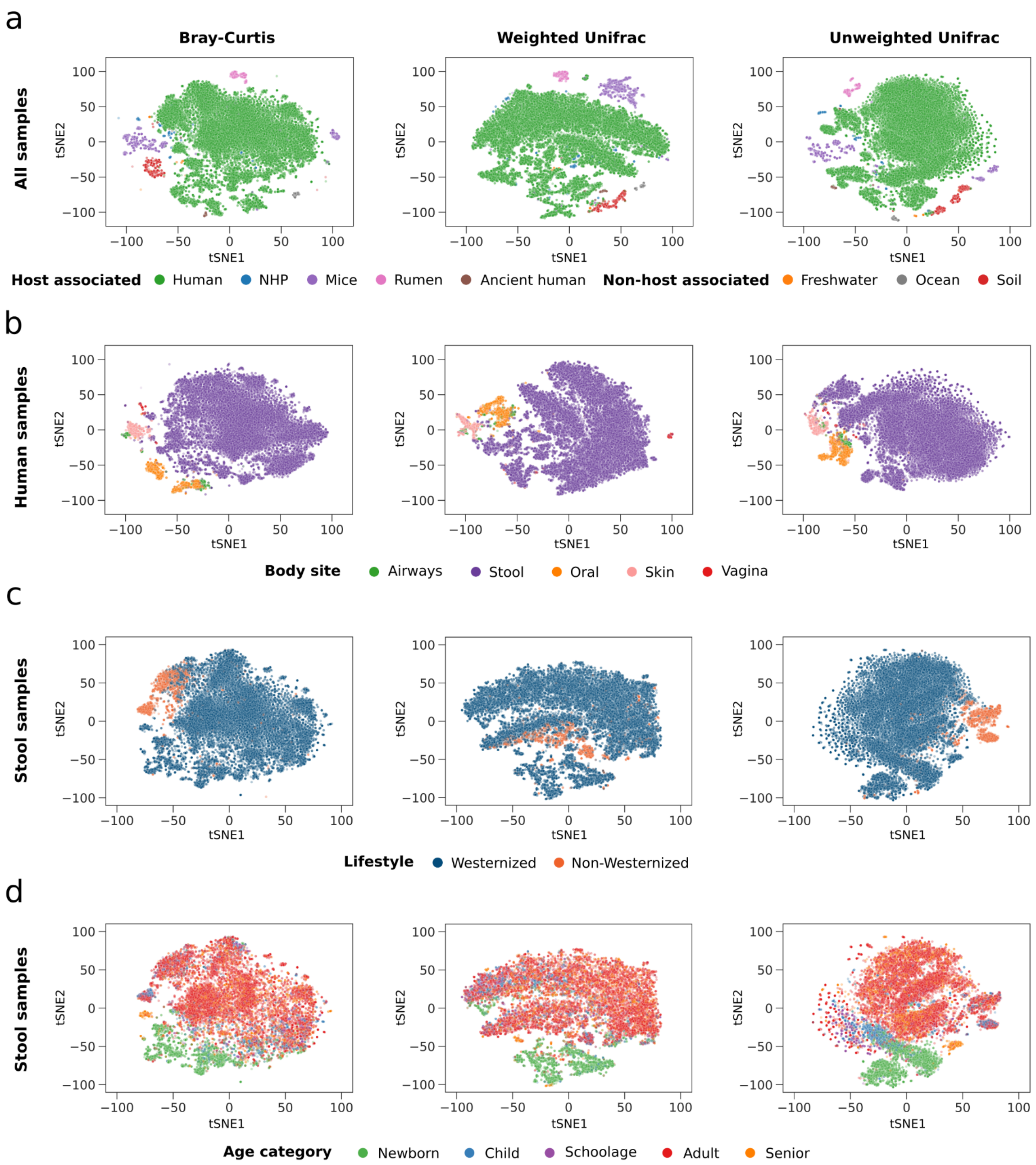

Supplementary Figure 4: t-SNE representation of the 24.5k metagenomic samples based on (from left to right) Bray-Curtis, Weighted Unifrac and Unweighted Unifrac distances of the MetaPhlAn 4 SGB-level taxonomic profiles. a, Dimensionality reduction using all 24.5k metagenomic samples classified by environment. **b,** Dimensionality reduction using only the 19.5k human metagenomic samples classified by body site. **c,** Dimensionality reduction using only modern human stool metagenomic samples classified by lifestyle. **d,** Dimensionality reduction using only modern human stool metagenomic samples classified by age category. NHP = Non-human primate.

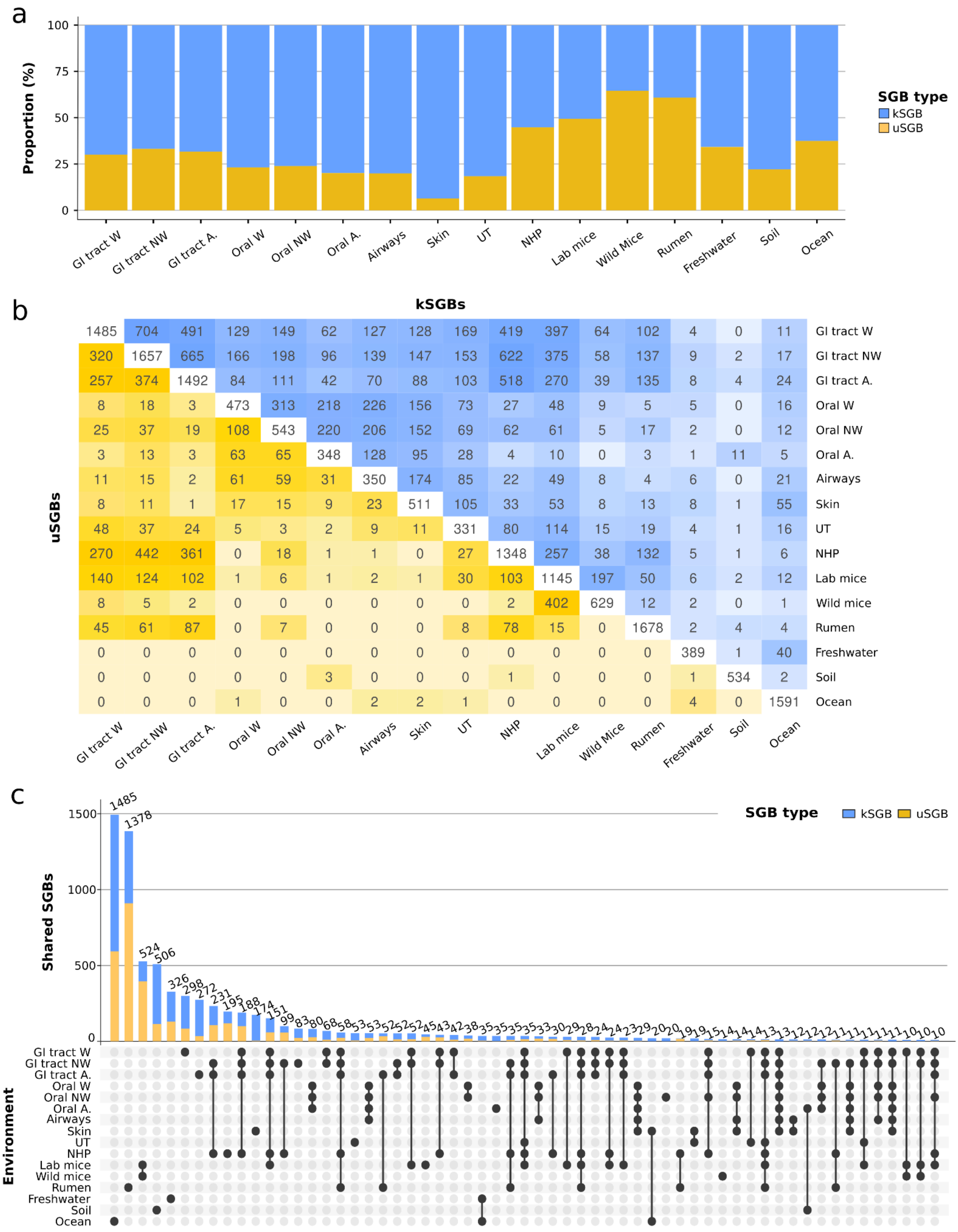

Supplementary Figure 5: Presence of SGBs across different environments. **a,** Proportion of the kSGBs and uSGBs across environments. **b**, Co-presence of SGBs across environments. The top triangular represents the number of kSGBs shared between each pair of environments, the bottom triangular shows the number of uSGBs shared and the diagonal contains the total number of SGBs detected in each individual environment. **c,** The upset plot represents the SGBs exclusively present in each group of environments. Only intersections with more than 10 SGBs are shown. GI=gastrointestinal, W=Westernized, NW=non-Westernized, NHP=non-human primates.

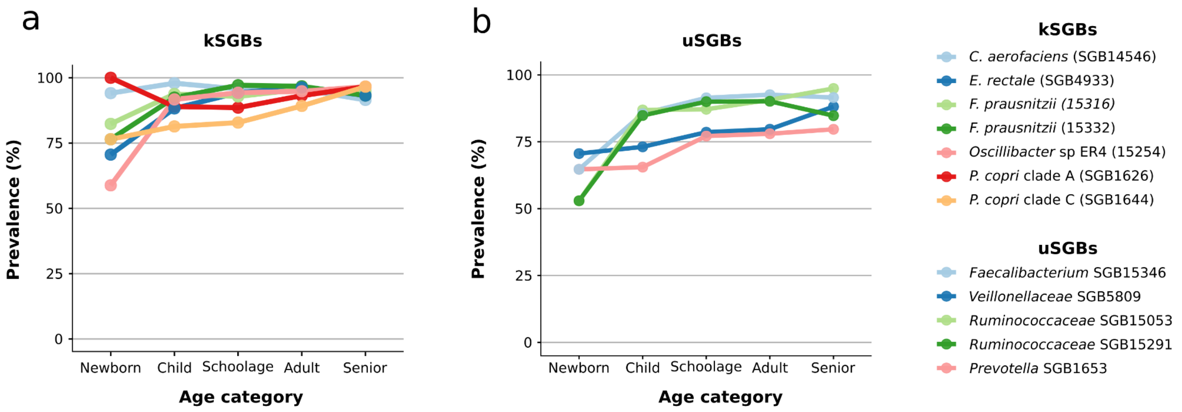

Supplementary Figure 6: Most prevalent known (left) and unknown (right) SGBs in the Non-Westernized populations and their abundance through life. The 2 most prevalent SGBs of each age category are shown.

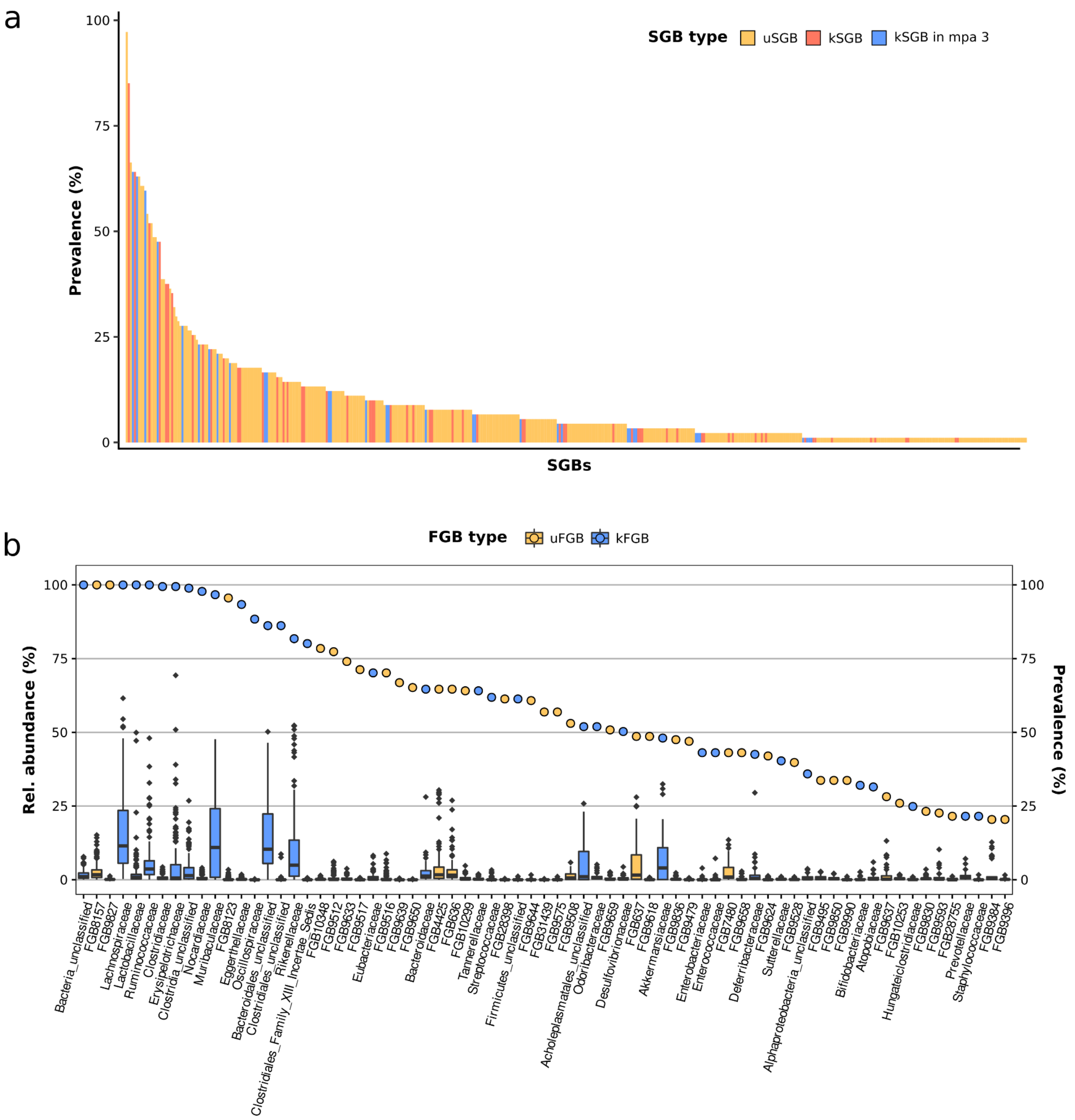

Supplementary Figure 7: Expanded microbial diversity of the mice gut microbiome. a, The MetaPhlAn 4 genomic database incorporates 3,678 MAGs from the Xiao, L. et al 2015 study [61] which span 437 different SGBs. The proportion of kSGB and uSGB based on these MAGs follows a distribution similar to that based on the MetaPhlAn 4 taxonomic profiles (**Fig. 3a**). **b,** Relative abundance and prevalence of the microbial families (FGBs) present in the mice gut (n=181 samples). FGBs present in more than 20% of the samples are shown. Box plots show the median (center), 25th/75th percentile (lower/upper hinges), 1.5× interquartile range (whiskers) and outliers (points).

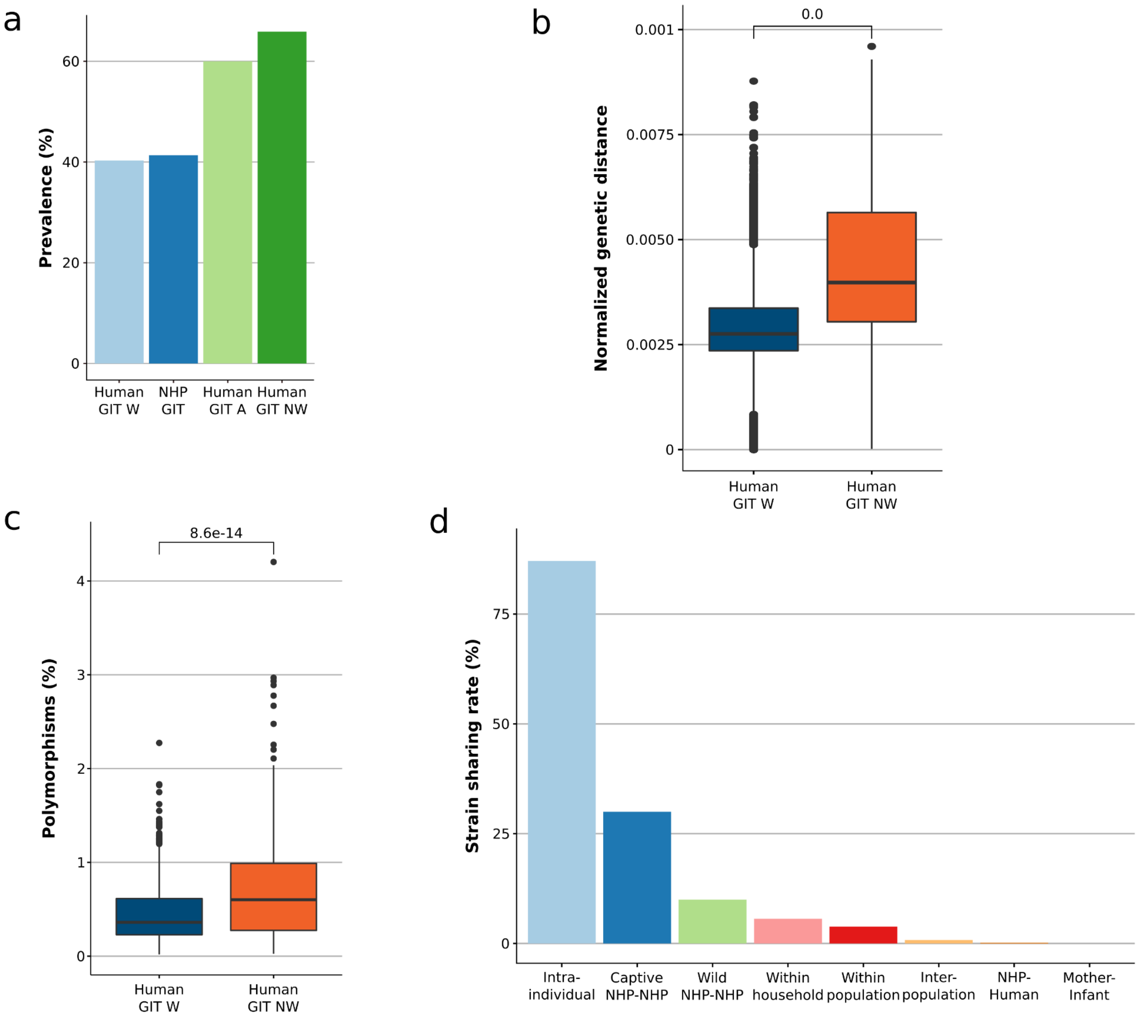

Supplementary Figure 8: Phylogenetic analysis of *Lachnospiraceae* SGB4894. a, Prevalence of *Lachnospiraceae* SGB4894 across different hosts. For the modern human gut, only samples from healthy individuals were selected. **b**, Intra-population normalized genetic distances differences between Westernized and non-Westernized populations (n=2,787 samples, two-sided Mann-Whitney U test = 0.0). **c**, Polymorphic rates differences between Westernized and non-Westernized populations (n=2,787 samples, two-sided Mann-Whitney U test = 8.6 e-14). **d,** Strain sharing rates of *Lachnospiraceae* SGB4894. GIT=gastrointestinal tract, W=Westernized, NW=non-Westernized, A=ancient, NHP=non-human primates. Box plots in **b** and **c** show the median (center), 25th/75th percentile (lower/upper hinges), 1.5× interquartile range (whiskers) and outliers (points).

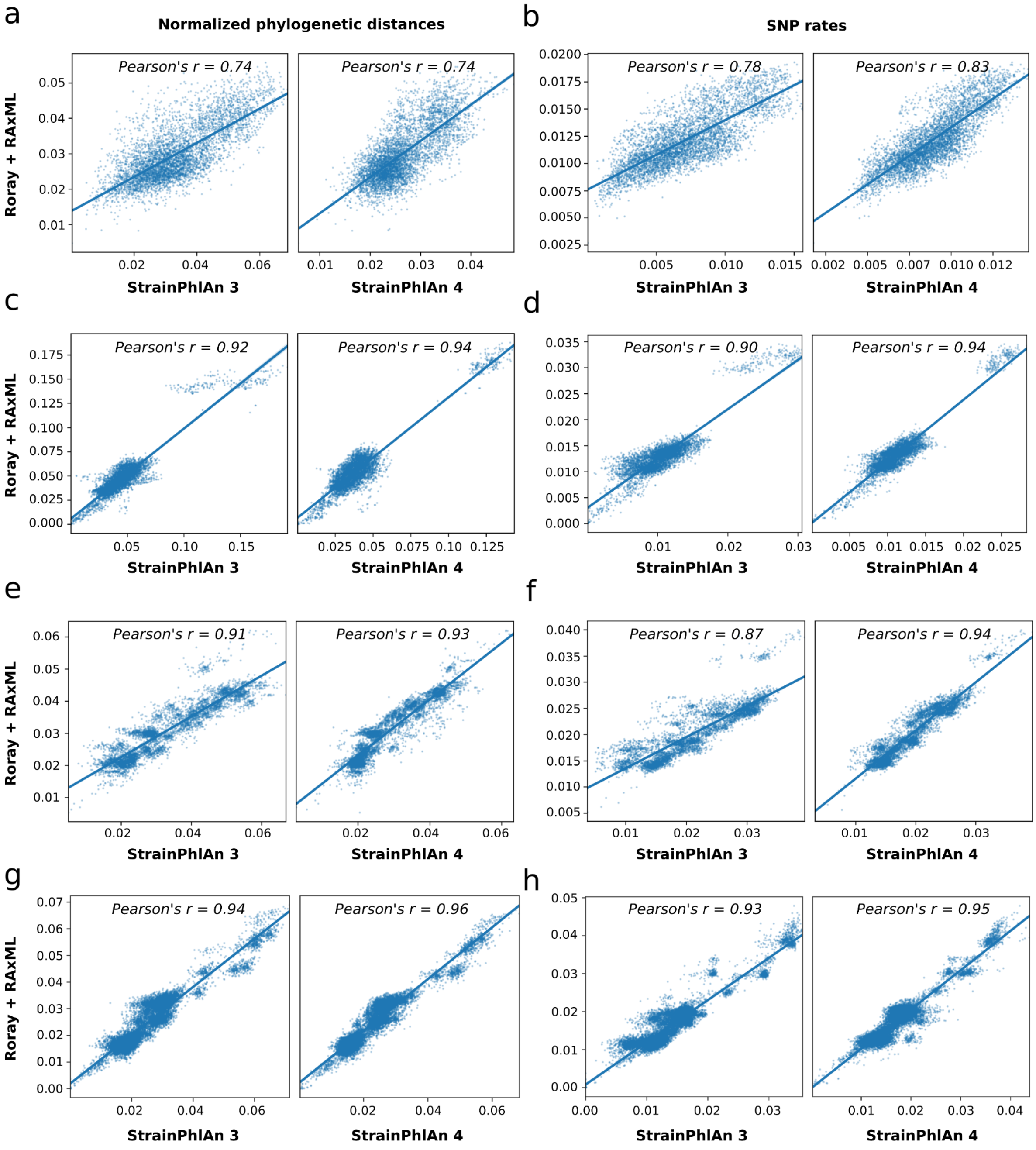

Supplementary Figure 9: Comparison between StrainPhlAn 3 and 4 phylogenetic reconstruction. Correlation of (**a,c,e,g**) the normalized pairwise phylogenetic distances (**b,d,f,h**) and SNP rates between the StrainPhlAn (x axis) and the Roary + RAxML (y axis) trees for (**a,b**) *Blautia wexlerae* (SGB4837), (**c,d**) *Bacteroides uniformis* (SGB1836), (**e,f**) *Eubacterium rectale* (SGB4933) and (**g,h**) *Lachnospiraceae* SGB4894.

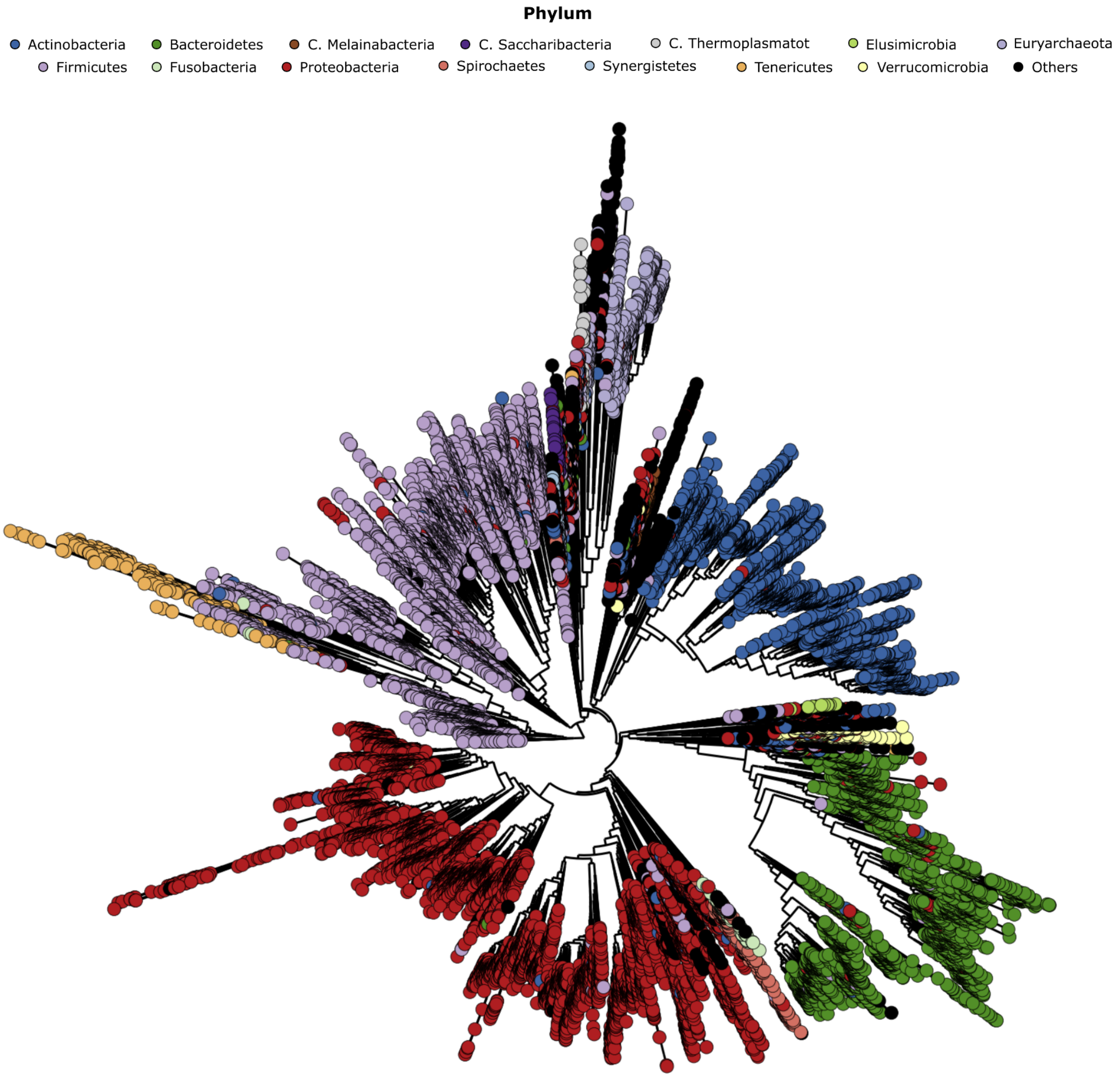

Supplementary Figure 10: MetaPhlAn 4 phylogenetic tree of life. Leaves color represent the assigned phylum of each of the 26,970 SGBs included in the database.

Supplementary Tables

Supplementary Table 1. SGB clustering results of the 729,195 genomes medium-to-high-quality genomes collected for this study
Supplementary Table 2. Catalog of the metagenomic assembled genomes employed for the database reconstruction (External file)
Supplementary Table 3. Catalog of the metagenomic samples employed in the MAG reconstruction
Supplementary Table 4. Distribution of phyla across SGBs in the genomic database
Supplementary Table 5. Evaluation of the species detection using the CAMI II and SynPhlAn synthetic metagenomes
Supplementary Table 6. Number of species detected by MetaPhlAn 4 in single-isolates datasets at different coverages . Results are expressed in terms of True positives / False positives
Supplementary Table 7. Evaluation of the relative abundance quantification performance using the CAMI II and SynPhlAn synthetic metagenomes
Supplementary Table 8. Differences in the percentage of randomly assigned reads between MetaPhlAn 3 and MetaPhlAn 4
Supplementary Table 9. Evaluation of the species detection using synthetic metagenomes containing kSGBs and uSGBs
Supplementary Table 10. Evaluation of the relative abundance quantification performance using synthetic metagenomes containing kSGBs and uSGBs
Supplementary Table 11. Full list of datasets employed in this study
Supplementary Table 12. Prevalence of SGB shared between all human body sites
Supplementary Table 13. Prevalence of SGBs across different environments
Supplementary Table 14. Prevalence of the human gut SGBs throughout life
Supplementary Table 15. Contemporary human gut metagenomic datasets employed in this study
Supplementary Table 16. Relative abundance of kSGB and uSGBs across age categories
Supplementary Table 17. Metagenomic samples used in the differential abundance analysis of diet-related taxa in the mice microbiome
Supplementary Table 18. Prevalence and relative abundance of the SGBs detected by MetaPhlAn 4 but not recovered from the MAGs in the XiaoL_2015 study
Supplementary Table 19. Results of the differential abundance analysis of diet-related taxa in the mice microbiome. Sex, age-in-days and genetic background were used as fixed effects, and the vendor as grouping variable. Significance was assessed via Wald-test and p-values were Benjamini-Hochberg corrected
Supplementary Table 20. Disease related studies employed for the Lachnospiraceae SGB4894 association analysis
Supplementary Table 21. Comparison between StrainPhlAn 3 and StrainPhlAn 4 multiple sequence alignments. The three most prevalent kSGBs and Lachnospiraceae SGB4894 were assessed
Supplementary Table 22. Ancient human and non-human primates gut metagenomic datasets used for the Lachnospiraceae SGB4894 strain-level analysis
Supplementary Table 23. Differences in the classified fraction of the reads between MetaPhlAn 3 and MetaPhlAn 4

Supplementary Table 24. Partial correlation between uSGBs and the panel of 19 representative cardiometabolic and nutritional markers from PREDICT1. Spearman's correlations were corrected for age, sex and body mass index. P-values were corrected through the Benjamini-Hochberg procedure

Supplementary Table 25. Metagenomic samples and MAGs selected for the StrainPhlAn evaluation
