# Supplementary material for: Extending and improving metagenomic taxonomic profiling with uncharacterized species using MetaPhlAn 4
Source: Nat Biotechnol. 2023 Feb 23;41(11):1633–44. doi: 10.1038/s41587-023-01688-w (PMC10635831; doi:10.1038/s41587-023-01688-w)
Supplement: Supplementary file 2 — Reporting Summary [file 41587_2023_1688_MOESM2_ESM.pdf]

nature portfolio

Corresponding author(s): Nicola Segata

Last updated by author(s): Jan 10, 2023

Reporting Summary

Nature Portfolio wishes to improve the reproducibility of the work that we publish. This form provides structure for consistency and transparency in reporting. For further information on Nature Portfolio policies, see our Editorial Policies and the Editorial Policy Checklist.

Statistics

For all statistical analyses, confirm that the following items are present in the figure legend, table legend, main text, or Methods section.

n/a	Confirmed	
☐	☒	The exact sample size (*n*) for each experimental group/condition, given as a discrete number and unit of measurement
☒	☐	A statement on whether measurements were taken from distinct samples or whether the same sample was measured repeatedly
☐	☒	The statistical test(s) used AND whether they are one- or two-sided<br>*Only common tests should be described solely by name; describe more complex techniques in the Methods section.*
☐	☒	A description of all covariates tested
☐	☒	A description of any assumptions or corrections, such as tests of normality and adjustment for multiple comparisons
☐	☒	A full description of the statistical parameters including central tendency (e.g. means) or other basic estimates (e.g. regression coefficient) AND variation (e.g. standard deviation) or associated estimates of uncertainty (e.g. confidence intervals)
☐	☒	For null hypothesis testing, the test statistic (e.g. F, t, r) with confidence intervals, effect sizes, degrees of freedom and P value noted<br>*Give P values as exact values whenever suitable.*
☒	☐	For Bayesian analysis, information on the choice of priors and Markov chain Monte Carlo settings
☒	☐	For hierarchical and complex designs, identification of the appropriate level for tests and full reporting of outcomes
☐	☒	Estimates of effect sizes (e.g. Cohen's *d*, Pearson's *r*), indicating how they were calculated

Our web collection on statistics for biologists contains articles on many of the points above.

Software and code

Policy information about availability of computer code

Data collection	No software was used for data collection.
Data analysis	Python v3.7, CheckM v1.1.4, MASH v2.0, PhyloPhlAn v3, fastcluster v1.1.25, Prokka v1.14, DIAMOND v0.9.24, UniRefAnnotator v1 (https://github.com/biobakery/uniref_annotator), UniRef release 2019_06, MMSeqs v2, Bowtie2 v2.3.5.1, GTDB-Tk v2.1.1, GTDB release 207, MetaPhlAn v3.0.12 & v4.beta.1, StrainPhlAn v3.0.12 & v4.beta.1, TrimAl v1.4.rev15, MAFFT v7.475, IQ-TREE v2.0.3, mOTUs v2.6, Bracken v2.5, OPAL v1.0.5, ART v2.5.8, curatedMetagenomicData v3, stats R v4.0.5, Roary v3.13.0, RAxML v8.2.4, PyPhlAn v1 (https://github.com/SegataLab/pyphlan), scikit-learn v0.22.2, ppcor v1.1, scipy v1.5.2, geosphere v1.5-10, GraPhlAn v1.1.4, scikit-bio v0.5.6, statsmodel v0.11.1, pheatmap v1.0.12

For manuscripts utilizing custom algorithms or software that are central to the research but not yet described in published literature, software must be made available to editors and reviewers. We strongly encourage code deposition in a community repository (e.g. GitHub). See the Nature Portfolio guidelines for submitting code & software for further information.

Data

Policy information about availability of data

All manuscripts must include a data availability statement. This statement should provide the following information, where applicable:

- Accession codes, unique identifiers, or web links for publicly available datasets
- A description of any restrictions on data availability
- For clinical datasets or third party data, please ensure that the statement adheres to our policy

All metagenomic studies analyzed in this work are publicly available through the corresponding publications listed in Supplementary Table 11. All reference genomes and taxonomic data are publicly available through the NCBI GenBank database (https://www.ncbi.nlm.nih.gov/genbank/). The GTDB release 207 is publicly available at https://gtdb.ecogenomic.org/. The CAMI II Challenge synthetic metagenomes and gold standards are available at https://www.microbiome-cosi.org/cami/cami/cami2. The SynPhlAn-nonhuman synthetic metagenomes and gold standards are available at http://segatalab.cibio.unitn.it/tools/biobakery. The novel synthetic metagenomes containing kSGBs and uSGBs and gold standards as well as the single-isolate synthetic metagenomes are available at http://segatalab.cibio.unitn.it/tools/metaphlan/. Prevalences of the SGBs across environments, age categories and lifestyles are available in Supplementary Tables 13-14. Metadata of the publicly analyzed human metagenomes is also available through the curatedMetagenomicData Rpackage 95. The full list of metagenomic studies used for the strain-level analysis of Lachnospiraceae SGB4894 is reported in Supplementary Tables 15 and 22.

Human research participants

Policy information about studies involving human research participants and Sex and Gender in Research.

Reporting on sex and gender	N/A
Population characteristics	N/A
Recruitment	N/A
Ethics oversight	N/A

Note that full information on the approval of the study protocol must also be provided in the manuscript.

Field-specific reporting

Please select the one below that is the best fit for your research. If you are not sure, read the appropriate sections before making your selection.

☐ Life sciences ☐ Behavioural & social sciences ☒ Ecological, evolutionary & environmental sciences

For a reference copy of the document with all sections, see nature.com/documents/nr-reporting-summary-flat.pdf

Ecological, evolutionary & environmental sciences study design

All studies must disclose on these points even when the disclosure is negative.

Study description	Here, we present a method - MetaPhlAn 4 - to integrate information from both metagenome assemblies and microbial isolate genomes for improved and more comprehensive metagenomic taxonomic profiling.
Research sample	To derive the database of SGBs we recovered 236,620 bacterial and archeal genomes available in NCBI and labeled as “reconstructed from isolate sequencing or single cells”. These were integrated with 771,528 MAGs assembled from samples collected from humans (5 distinct main human body sites, 164 distinct human cohorts), animal hosts (including 22 non-human primate species), and non-host associated environments (including soil, fresh water, and oceans, Tables S2 and S3). To assess the improvements of the tool, we also recovered a total of 24,515 metagenomic samples (145 distinct studies, Table S8) from different human, animal, and non-host associated environments.
Sampling strategy	No sampling was performed.
Data collection	All data from NCBI and GTDB was obtained using their respective publicly available FTP sites. Synthetic metagenomes from CAMI II challenge and the BioBakery 3 were downloaded from their respective web sites. No software was used for data collection.
Timing and spatial scale	All data was downloaded on November 2020.
Data exclusions	Genomes were quality filtered based on their completeness (>50%) and contamination (<5%).
Reproducibility	All the code employed in the study is deterministic. All the analyses shown in the manuscript can be reproduced following the Methods section.

Randomization	No randomization was performed.
Blinding	No blinding was performed.

Did the study involve field work? ☐ Yes ☒ No

Reporting for specific materials, systems and methods

We require information from authors about some types of materials, experimental systems and methods used in many studies. Here, indicate whether each material, system or method listed is relevant to your study. If you are not sure if a list item applies to your research, read the appropriate section before selecting a response.

Materials & experimental systems

n/a	Involved in the study
☒	☐ Antibodies
☒	☐ Eukaryotic cell lines
☒	☐ Palaeontology and archaeology
☒	☐ Animals and other organisms
☒	☐ Clinical data
☒	☐ Dual use research of concern

Methods

n/a	Involved in the study
☒	☐ ChIP-seq
☒	☐ Flow cytometry
☒	☐ MRI-based neuroimaging
